# CRISPR Technology in Disease Management: An Updated Review of Clinical Translation and Therapeutic Potential

**DOI:** 10.1111/cpr.70099

**Published:** 2025-07-20

**Authors:** Bahareh Farasati Far, Marziyeh Akbari, Mohammad Amin Habibi, Morteza Katavand, Sherko Nasseri

**Affiliations:** ^1^ Research Laboratory of Green Organic Synthesis and Polymers, Department of Chemistry Iran University of Science and Technology Tehran Iran; ^2^ Department of Genetics, Faculty of Science Shahrekord University Shahrekord Iran; ^3^ Gene, Cell & Tissue Research Institute, Tehran University of Medical Science Tehran Iran; ^4^ Clinical Research Development Center, Qom University of Medical Sciences Qom Iran; ^5^ Department of Genetics, Faculty of Basic Science Islamic Azad University, Science and Research Branch Tehran Iran; ^6^ Department of Neuroscience and Regenerative Medicine Medical College of Georgia, Augusta University Augusta Georgia USA

**Keywords:** cancers, CRISPR‐Cas systems, epigenome editing, genetic therapy, iPSC, molecular diagnostic techniques, nervous system diseases, personalised medicine

## Abstract

CRISPR‐Cas9 technology has rapidly advanced as a transformative genome‐editing platform, facilitating precise genetic modifications and expanding therapeutic opportunities across various diseases. This review explores recent developments and clinical translations of CRISPR applications in oncology, genetic and neurological disorders, infectious diseases, immunotherapy, diagnostics, and epigenome editing. CRISPR has notably progressed in oncology, where it enables the identification of novel cancer drivers, elucidation of resistance mechanisms, and improvement of immunotherapies through engineered T cells, including PD‐1 knockout CAR‐T cells. Clinical trials employing CRISPR‐edited cells are demonstrating promising results in hematologic malignancies and solid tumours. In genetic disorders, such as hemoglobinopathies and muscular dystrophies, CRISPR‐Cas9 alongside advanced editors like base and prime editors show significant potential for correcting pathogenic mutations. This potential was affirmed with the FDA's first approval of a CRISPR‐based therapy, Casgevy, for sickle cell disease in 2023. Neurological disorders, including Alzheimer's, ALS, and Huntington's disease, are increasingly targeted by CRISPR approaches for disease modelling and potential therapeutic intervention. In infectious diseases, CRISPR‐based diagnostics such as SHERLOCK and DETECTR provide rapid, sensitive nucleic acid detection, particularly valuable in pathogen outbreaks like SARS‐CoV‐2. Therapeutically, CRISPR systems target viral and bacterial genomes, offering novel treatment modalities. Additionally, CRISPR‐mediated epigenome editing enables precise regulation of gene expression, expanding therapeutic possibilities. Despite these advances, significant challenges remain, including off‐target effects, delivery methodologies, immune responses, and long‐term genomic safety concerns. Future improvements in editor precision, innovative delivery platforms, and enhanced safety assessments will be essential to fully integrate CRISPR‐based interventions into standard clinical practice, significantly advancing personalised medicine.

## Graphical abstract

CRISPR‐Cas systems offer transformative genome editing capabilities for precise manipulation of cellular genes. This enables two main therapeutic avenues: ex vivo modification of patient cells for re‐transplantation or direct in vivo gene targeting via advanced delivery methods. These approaches unlock significant potential for developing targeted therapies across diverse diseases, advancing personalised medicine.

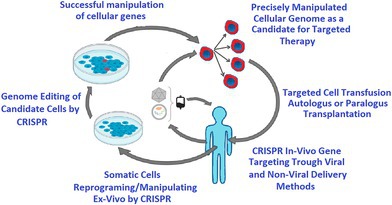

**Abbreviations**
AAV: adeno‐associated virus
ABE: adenine base editors
ABL: abelson murine leukemia viral oncogene homologue 1
AD: Alzheimer Disease
AHC: adrenal hypoplasia congenital
ALS: amyotrophic lateral sclerosis
AML: acute myeloid leukemia
APOBEC: apolipoprotein B mRNA editing catalytic polypeptide‐like
APP: amyloid precursor protein
ATTR: amyloid polyneuropathy, transthyretin‐related
BCR: B cell receptor
BE: base editors
BLISS: breaks labelling in situ and sequencing
BRAF: B‐Raf proto‐oncogene, serine/threonine kinase
CAR: chimeric antigen receptor
CasMINI: a compact, miniature CRISPR‐Cas system (e.g., a small Cas12f variant)
CBE: cytosine base editors
CF: cystic fibrosis
CLAN: CRISPR loci annotation (i.e., a tool or nomenclature for annotating CRISPR loci)
CLL: chronic lymphocytic leukaemia
CML: chronic myeloid leukaemia
CRC: colorectal cancer
CRISPR: clustered regularly interspaced short palindromic repeats
CVD: cardiovascular disease
DETECTR: DNA endonuclease targeted CRISPR trans reporter
DLBCL: diffuse large B‐cell lymphoma
DMD: Duchenne muscular dystrophy
DMT: DNA methyl transferase
DREBIC: drug response evaluation by in vivo CRISPR
DS: down syndrome
DrS: Dravet syndrome
DSB: double‐strand break
EBV: Epstein–Barr virus
EGFR: epidermal growth factor receptor
EMT: epithelial‐to‐mesenchymal transition
ES: embryonic stem
EV: extracellular vesicles
FA: Fanconi anaemia
FDA: food and drug administration
FL: follicular lymphoma
FXS: fragile X syndrome
HBB: haemoglobin beta
HCC: hepatocellular carcinoma
HD: huntington's disease
HDR: homology‐directed repair
HH: hypogonadotropic hypogonadism
HHL: hereditary hearing loss
HIV: human immunodeficiency virus
HPV: human papillomavirus
HR: homologous recombination
HSC: haematopoietic stem cells
HT1: hereditary tyrosinemia type I
IE: immediate‐early
KRAB: Krüppel‐associated box
LHNP: lipid hybrid nanoparticles
MCL: mantle cell lymphoma
MEK: mitogen‐activated protein kinase kinase
MeSH: medical subject headings
MICA: MHC class I polypeptide‐related sequence A
MMEJ: microhomology‐mediated end joining
MND: motor neuron disease
NHEJ: non‐homologous end joining
NHGRI: national human genome research institute
NK: natural killer
OS: overall survival
PAM: protospacer‐adjacent motif
PARP: poly ADP‐ribose polymerase
PD: Parkinson's disease
PE: prime editors
PKU: phenylketonuria
PPC: patient‐derived primary cells
PRISMA: preferred reporting items for systematic reviews and meta‐analyses
PV: polycythemia vera
RecQ: RecQ helicase
RESCUE: RNA editing for specific c‐to‐u exchange
RNA: ribonucleic acid
RNP: ribonucleoprotein particle
RP: retinitis pigmentosa
RPA: replication protein A
SCD: sickle cell disease
SCID: severe combined immune deficiency
SMA: spinal muscle atrophy
SMN: survival‐motor neuron
SSA: single strand annealing
TALEN: transcription activator‐like effector nucleases
TGFR: transforming growth factor beta receptor
UCD: urea cycle disorder
VEGFA: vascular endothelial growth factor A
ZFN: zinc‐finger nucleases

## 1 Background

CRISPR was first discovered by Ishino et al., and later characterised by Doudna and Charpentier [1, 2]. The CRISPR sequences are regarded as the adaptive immune systems in many bacteria and archaea to protect them from bacteriophage, viral DNA, and plasmid invasion [3, 4]. The CRISPR‐Cas9 system consists of the Cas9 endonuclease and a guide RNA (gRNA) [1, 5], which scans the genome for a complementary sequence adjacent to a protospacer adjacent motif (PAM) [6, 7]. Once the PAM is recognised, Cas9 creates a double‐strand break (DSB) upstream of the PAM site [8, 9].

DNA damage activates repair pathways such as non‐homologous end joining (NHEJ) and homology‐directed repair (HDR) [2, 10] NHEJ, a template‐free mechanism, joins broken DNA ends, often resulting in small insertions or deletions that can cause gene knock‐outs. HDR, effective in the S and G2 phases, uses a homologous sequence for precise repair and allows for gene correction or knock‐ins [11, 12, 13, 14] Gene knock‐outs are more efficient due to the limited activation of HDR during certain cell cycle stages [15].

The CRISPR‐Cas system can precisely edit target DNA, offering potential treatments for genetic diseases and various refractory conditions, from sickle cell anaemia to cancer [16, 17]. Despite global research efforts, technical challenges in treating genetic diseases persist. This review examines the CRISPR‐Cas molecular mechanism, types, applications, and strategies in personalised medicine, along with associated challenges and limitations.

## 2 Method

### 2.1 Objective and Search Strategy

This study investigates CRISPR‐Cas technology's potential in treating challenging diseases by examining pre‐clinical and clinical studies to highlight its efficacy in managing difficult‐to‐treat disorders. A comprehensive literature search was conducted using databases such as PubMed, Web of Science, and Google Scholar. Keywords included ‘CRISPR Technology’, ‘Genetic Therapy’, ‘Clinical Applications’, ‘Delivery Methods’, ‘Monogenic disorders’, ‘Neurological disorders’, ‘Infectious diseases’, ‘Immunotherapy’, ‘Epigenetic editing’, The search covered articles published between 2010 and 2023.

### 2.2 Eligibility Criteria

- English studies
- Studies included in this review were original research articles, clinical trials, and review articles that focused on the application of CRISPR technology in treating diseases between 2010 and 2023.
- Studies reporting CRISPR technology to treat challenging diseases, including cancers, neurodegenerative disorders, genetic diseases, infectious diseases, and autoimmune disorders
- Studies report on the use of personalised medicine approaches for treating challenging diseases
- Studies reporting on the clinical applications of CRISPR technology and personalised medicine in human patients
- Studies reporting on the safety, efficacy, and feasibility of CRISPR technology and personalised medicine approaches

#### 2.2.1 Exclusion Criteria

- Non‐English studies
- Studies outside the period between 2010 and 2023
- Studies that did not report on the use of CRISPR technology or personalised medicine to treat challenging diseases
- Studies that did not report the clinical applications of CRISPR technology or personalised medicine in human patients
- Articles not available in English, studies not involving CRISPR‐Cas systems, and papers with insufficient methodological details
- The authors assessed studies that were not peer reviewed or of low quality.

### 2.3 Study Selection

Two reviewers (M.A.H and M.A) conducted the study selection process independently according to the eligibility criteria. The screening process was performed by EndNote V.20 as follows: after yielding the initial articles, (1) the duplicate articles were removed, (2) the bi‐step title abstract process was done, and (3) a full‐text assessment was performed. Finally, studies that fully met the eligibility criteria were considered for data synthesis. If two reviewers disagree, a third senior reviewer (B.F.) makes the final decision.

## 3 Results

**FIGURE 1:**
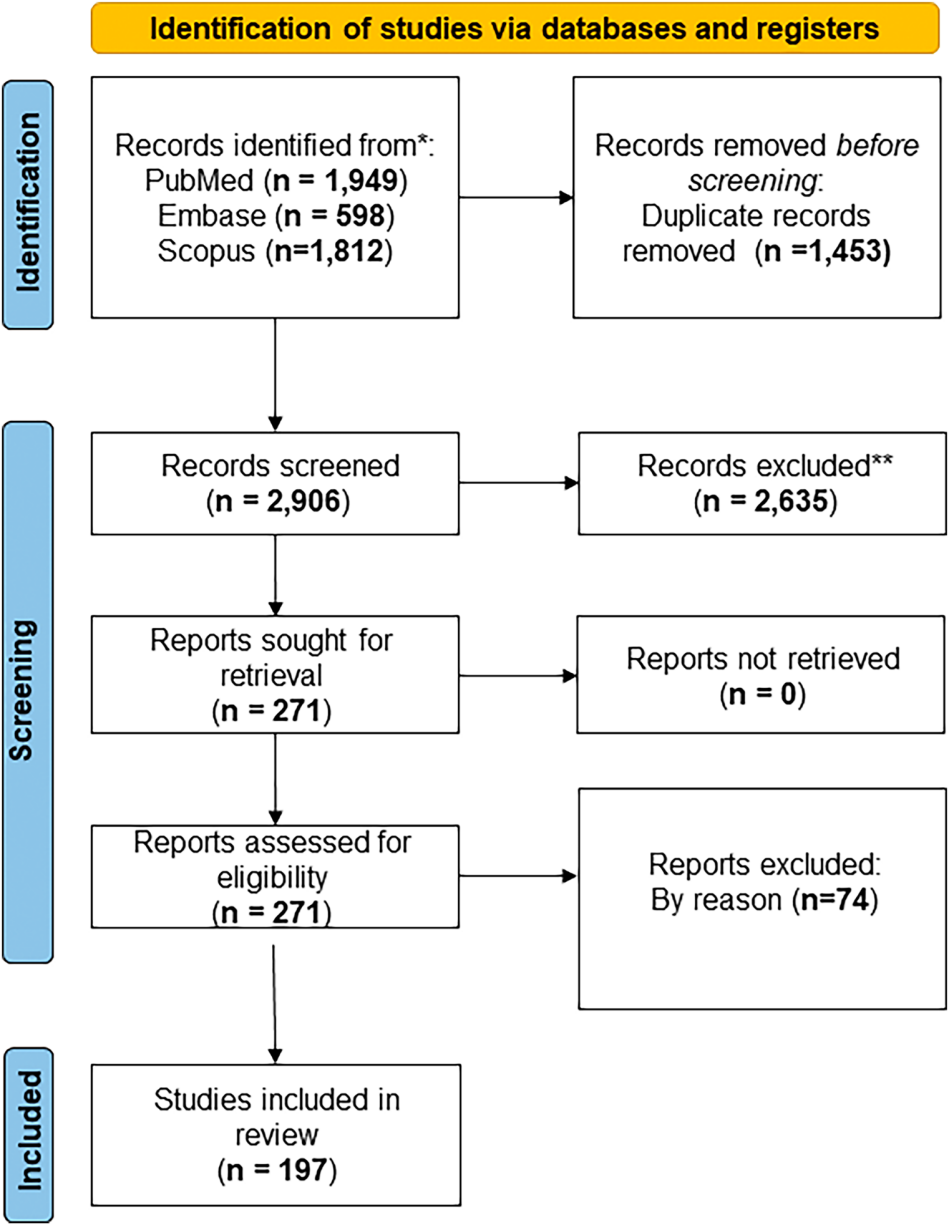
PRISMA flowchart of the screening process.

The search was conducted and yielded 4359 articles. After removing duplicates (*n* = 1453), 2906 articles were screened based on their title and abstract for relevance. Full‐text articles were then retrieved for the remaining 271 articles and assessed for eligibility according to the inclusion and exclusion criteria. Finally, 197 articles were included for data synthesis within the context. Figure 1 represents the PRISMA flowchart of the screening process. The included studies demonstrated the potential of CRISPR technology in various therapeutic areas. Key findings include successful applications in cancer immunotherapy, correction of genetic mutations in inherited diseases, and the development of CRISPR‐based diagnostics for infectious diseases. The majority of studies reported high specificity and efficacy of CRISPR systems, though challenges such as off‐target effects and delivery methods remain significant hurdles.

### 3.1 Molecular Aspects of CRISPR

#### 3.1.1 Molecular Mechanism of CRISPR Structure and Function

The CRISPR‐Cas9 system comprises two main components: a guide RNA (gRNA) and a CRISPR‐associated (Cas) nuclease. The gRNA is an RNA molecule that recognises and binds precisely to the target DNA, guiding the Cas nuclease to cut or edit [18, 19]. Ribonucleoprotein (RNP)‐mediated CRISPR genome editing, which involves the Cas9 protein and a single guide RNA (sgRNA), has advantages such as higher efficiency, improved cell viability, ease of transfer, and avoidance of plasmid‐related issues. The Cas nucleases are classified into Type 1 and Type 2, with six subdivisions [20] (Table 1). The gRNA is composed of two parts: the CRISPR RNA (crRNA), which contains a 17–20 nucleotide sequence that is complementary to the target DNA, and a tracer RNA that is 80 nucleotides long and binds to the crRNA, serving as a scaffold for the Cas nuclease [21]. The PAM is a short specific sequence located immediately after the complementary DNA sequence, and it is crucial for Cas nuclease cleavage. The PAM, approximately 2–6 nucleotides long, is positioned on the non‐target strand downstream of the targeted DNA sequence, directing the Cas nuclease to cleave 3–4 nucleotides upstream of it (Table 1) [7, 22].

**TABLE 1:** General classification of CRISPR‐Cas system‐based Cas protein type, PAM site, and target sequence.

	Cas type	PAM site	Structure and signature	Target
Cas protein Class 1	Type I (subtype A–G)	I‐A: 5‐CCN‐3 I‐B: 5‐TTC ACT‐3 5‐TAA −3 5‐TAT‐3 5‐TAG‐3 5‐ACA‐3 I‐C: 5‐NTTC‐3 I‐U: NA I‐D: NA I‐E: 5‐AAG‐3 I‐F: 5‐GCGC‐3	Multi subunit Cas protein; Signature protein: I: Cas3	Single strand DNA
	Type III (subtype A–D)	Not recognised	Multi subunit Cas protein; Signature protein: III: cas10	DNA/RNA
	Type IV (subtype A–C)	Not recognised	Multi subunit Cas protein; Signature protein: IV: Csf1	Not–recognised
Cas protein Class 2	Type II (subtype A–C)	5‐NGG‐3	Single subunit Signature protein: II: Cas9	DNA
	Type V (subtype A–I, K)	V‐A: 5‐TTTN‐3 V‐B: 5‐TTT‐3 5‐TTA‐3 5‐TTC‐3 V‐C & V‐D: 5‐AT rich PAM V‐E: 5‐TTCN‐3 V‐U 1 to 5 Not recognised	Single subunit Signature protein: II: Cas12 Cas14(12f)	Cas12: dsDNA/ssDNA Cas14: ssDNA
	Type VI (subtype A–C)	VI‐A: Non‐G (predicted) VI‐B1 & B2: NAN/NAA‐3 (Predicted)	Signature protein: Signature protein: II: Cas13	Single strand RNA
	Type I (subtype A–G)	I‐A: 5‐CCN‐3 I‐B: 5‐TTC ACT‐3 5‐TAA −3 5‐TAT‐3 5‐TAG‐3 5‐ACA‐3 I‐C: 5‐NTTC‐3 I‐U: NA I‐D: NA I‐E: 5‐AAG‐3 I‐F: 5‐GCGC‐3	Multi subunit Cas protein: Signature protein: I: Cas3	Single strand DNA

*Note*: The CRISPR‐Cas system has revolutionised genetic engineering, allowing for precise and efficient manipulation of genetic material. The Cas proteins play a crucial role in this process by recognising and binding to specific DNA sequences, thereby facilitating targeted editing.

#### 3.1.2 CRISPR‐Cas9 Mediated Repairing System

**FIGURE 2:**
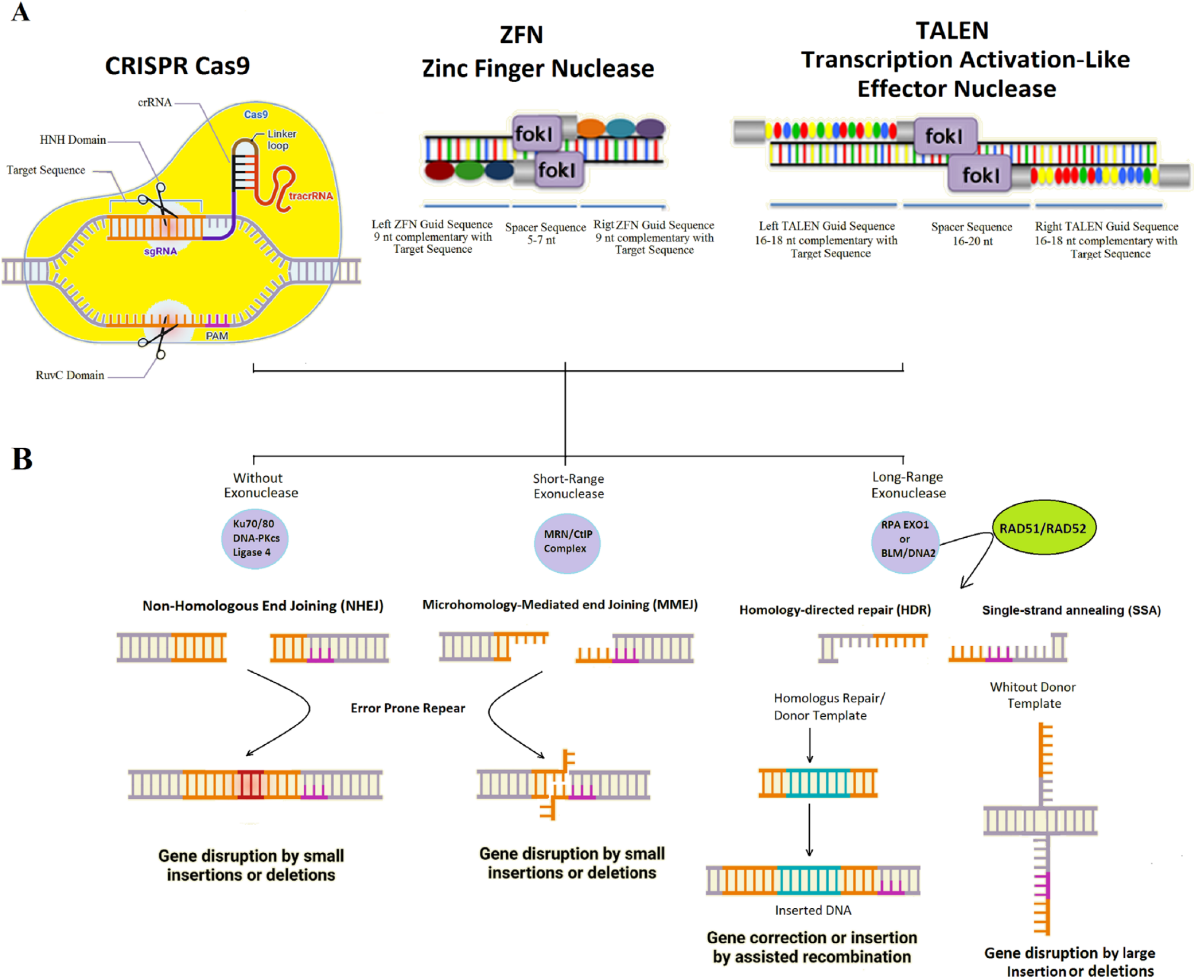
CRISPR‐Cas9, ZFN, and TALEN as genome editing tools. (A) CRISPR‐Cas9, through a single guide RNA complemented to target DNA and Cas9 protein creates a double‐stranded DNA cut. ZFN and TALEN‐engineered DNA‐binding proteins fused with the restriction enzyme FOK1 facilitate targeted genome editing by creating DNA double‐strand breaks. (B) The broken DNA will undergo the repair pathways: NHEJ or HDR; DSBs are repaired by NHEJ directly ligating the broken ends together with minimal DNA end processing when the ring‐shaped Ku70/Ku80 protein heterodimer binds to the DSB ends, which protects the DNA ends from exonucleases and also recruits the DNA‐dependent protein kinase and DNA ligase subsequently. Like NHEJ, MMEJ does not require a template for repairing the damaged DNA and begins with short‐range resection of the DSB. In MMEJ, DSB ends are re‐aligned using overhanged microhomologous sequences, and further the remaining 3’ ssDNA flaps are cleaved off, which results in small indels. ssDNA overhangs (> 1000 nucleotides) are generated by the 5′–3′ exonuclease activities of either EXO1 or BLM‐DNA2, and then coated by RPA, followed by the binding of RAD52, which mediates SSA pathway. Unless, RPA‐ssDNA can serve as a substrate for the RAD51 filament assembly, allowing for repair by HDR.

Unlike the effector complexes of Meganucleases, ZFNs, and TALENs, which rely on protein‐DNA interactions, the CRISPR‐Cas system leverages RNA–DNA base pairing and protein‐DNA interactions (Figure 2A) [23]. Furthermore, because of the long target sequence of CRISPR‐Cas (> 20 nucleotides) in comparison with Meganucleases, ZFNs, and TALENs (< 20 nucleotides), the CRISPR‐Cas system has been more precise and effective than others [21, 24]. Genome editing technologies that manipulate genome sequences take advantage of endonucleases to induce DS DNA DSBs at desired locations in the genome [25]. The processing of DSBs by the cellular DSB repair machinery is a determining factor in the type and success of gene manipulation outcomes. Therefore, the accuracy and efficiency of genome editing are influenced by the cellular DSB repair pathways [26]. Double‐strand breaks (DSBs) pose a threat to genome integrity, and cells have evolved multiple repair mechanisms. Therefore, the cellular DSB repair pathways influence the accuracy and efficiency of genome editing [27]. In Figure 2B, the CRISPR‐Cas9 system and the mechanisms of DSB repair are briefly depicted. The cell picks the particular repair pathway depending on different species, the cell cycle, target site sequence, and chromatin structure. Overall, mammalian cells intrinsically choose cNHEJ throughout the cell cycle, although HR and SSA are more frequently utilised during the S/G2 phase [28, 29].

#### 3.1.3 CRISPR Variants‐Based Target Modifications

**FIGURE 3:**
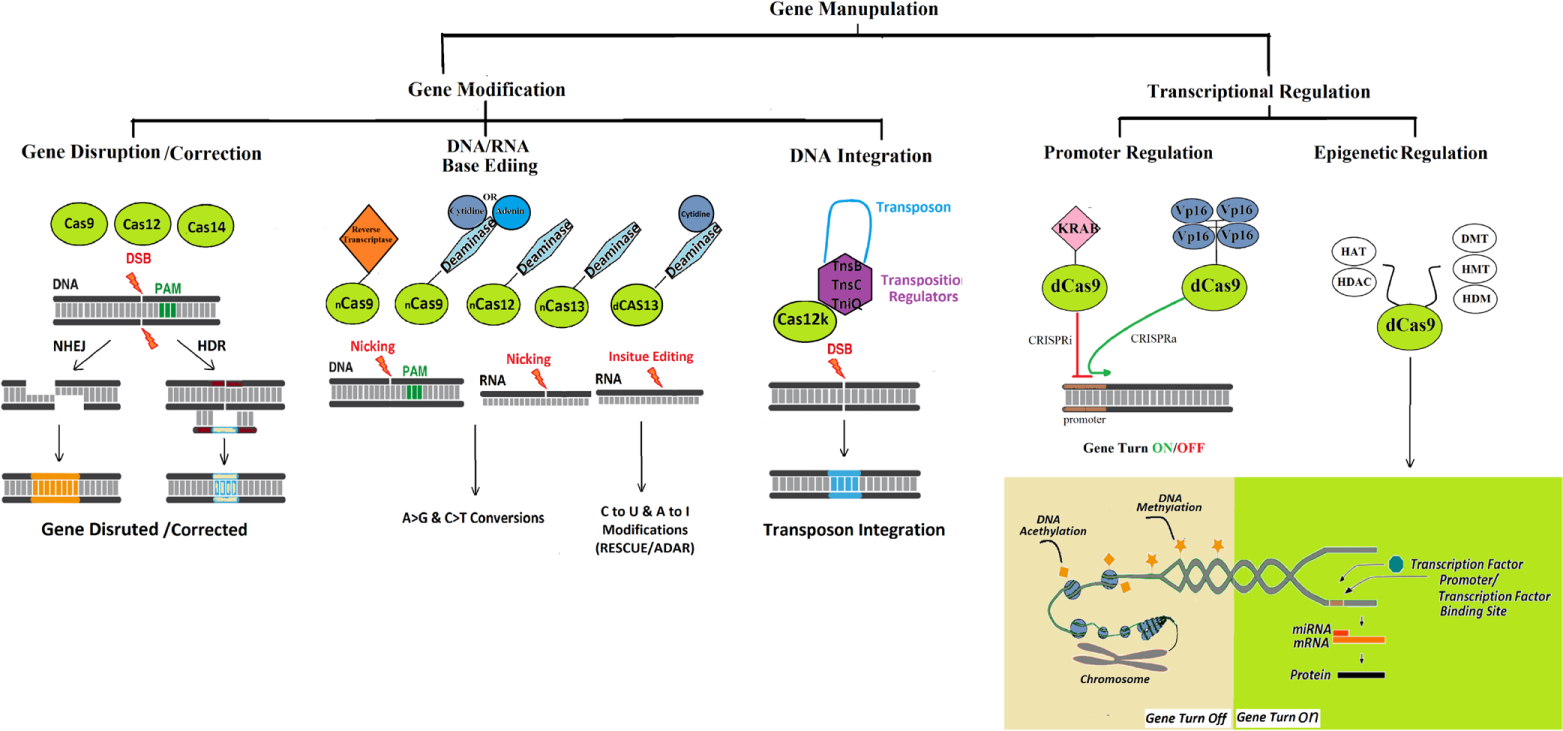
CRISPR‐Cas system strategies for gene manipulation via sequence editing, DNA integration, and transcriptional regulation. This figure illustrates multiple CRISPR‐based strategies for nucleotide and gene expression modification. Gene disruption or correction is achieved by CRISPR nucleases such as Cas9, Cas12, and Cas14, which introduce double‐strand breaks (DSBs) at target DNA sites, subsequently repaired by non‐homologous end joining (NHEJ) or homology‐directed repair (HDR). DNA and RNA base editing is performed using catalytically impaired Cas proteins (nCas9, nCas12, dCas13) fused to deaminase enzymes, allowing A‐to‐G or C‐to‐T conversions in DNA (by CBEs and ABEs) and reversible C‐to‐U or A‐to‐I conversions in RNA (via RESCUE or ADAR fusion systems). Targeted transposon integration is enabled by Cas12k in complex with transposase components such as TnsB, TnsC, and TniQ. Transcriptional regulation is mediated by dCas9 fused to transcriptional effectors such as KRAB (repression, CRISPRi) or VP16/VP64 (activation, CRISPRa), modulating gene expression at the promoter level. Finally, epigenetic modulation is accomplished by fusing dCas9 to chromatin‐modifying enzymes including histone acetyltransferases (HATs), deacetylases (HDACs), DNA methyltransferases (DMTs), and histone methylation/demethylation regulators (HMTs, HDMs), enabling locus‐specific and heritable gene expression changes.

DNA or RNA sequence modification based on CRISPR is performed using different enzymes such as Cas9, Cas12, and Cas14, and also the complexes of fused enzymes under the guidance of guide RNAs. Single‐strand or double‐strand DNA cleavage using these enzymes triggers repair processes, leading to gene disruption and indel mutations through NHEJ, or precise sequence modification can be achieved through HDR in the presence of template DNA [30, 31]. The fusion of Cas9 nickase (nCas9) or catalytically inactivated Cas9 (dead Cas9 or dCas9), which has been fused with functional domains of base editing enzymes, can also be used for genetic and epigenetic modifications, including base editing, to specific DNA sequences [32] (Figure 3). The base editors (BEs) comprise a cytidine deaminase like APOBEC mediated by dCas9, and nCas9 acts. This complex acts as an in situ modification, which in the case of dCas9 does not require the double‐strand breaks, while nCas9 is used to target specific loci through nick generation [33]. There are two prominent base editors: cytidine base editors (CBEs), which allow C to T conversions, and adenine base editors (ABEs), which enable A to G conversions. Additionally, the ADAR enzyme (Adenosine deaminases acting on RNA) fused with dCas13 for reversible and temporal A to G conversion (RNACas13‐ADAR) in fusions enables targeted editing of RNA [34, 35]. The transposase‐CRISPR‐mediated targeted integration can be induced by recruiting transposase complexes along with the guidance of catalytically dead Cas12k, resulting in targeted integration of gene fragments into the genome as a homology‐independent knock‐in strategy [36] (Figure 3).

dCas13 has been used for a wider variety of transcriptome regulation applications in addition to its well‐established function in ADAR‐mediated A‐to‐I RNA editing. For example, the RESCUE system (RNA Editing for Specific C‐to‐U Exchange) couples dCas13 with a cytidine deaminase domain to convert cytidine (C) to uridine (U) in target RNAs, thereby expanding the RNA editing toolkit beyond A‐to‐I modifications [37]. Furthermore, non‐coding RNAs, such as long non‐coding RNAs and microRNAs, can be functionally regulated using dCas13‐based platforms that target RNA molecules without altering the underlying genomic DNA [38]. Catalytically inactive Cas13 (dCas13) can be directed to specific RNA transcripts to modulate gene expression post‐transcriptionally—either by repressing translation through steric hindrance of ribosome binding or by enhancing expression via fusion to translation initiation factors [39, 40]. This RNA‐level regulation is transient and reversible, offering a significantly lower risk of long‐term off‐target effects compared to DNA editing methods, making it an attractive strategy for therapeutic and research applications [38] (Figure 3).

Prime Editing is a genome editing technique that enables precise DNA insertions, base substitutions, and specific deletions using a mechanism that functions without DSBs and donor templates [41]. Prime Editing achieves its operations by integrating Cas9 nickase and reverse transcriptase elements into one protein complex that utilises pegRNA for direct DNA modification guidance [41, 42]. By extending editing operations and shielding the genome from unnecessary mutations, prime editing produces a precise tool with adaptable genome modification capabilities that can be used for a range of therapeutic purposes(Figure 3).

The CRISPRa and CRISPRi variants can either stimulate or inhibit gene expression, depending on the attached regulatory region. The dCas9‐KRAB complex mediates transcriptional repression of genes within their endogenous genomic loci, while the dCas9‐VP64version of CRISPRa was developed by fusing four VP16 units (VP64) and regarded as a solid transcriptional activator [43, 44]. Site‐specific gene regulation can be achieved by altering DNA structure or histone modifications. CRISPR‐mediated epigenetic editing has shed light on allele‐specific epigenome editing, which utilises inactive dCas9 as a DNA‐binding domain and fused enzymes such as DNA methyl transferase (DMT), histone methyl transferase(HMT), histone demethylase(HDM), acetyltransferase(HAT), and deacetylases(HDAC) that can be targeted to alter the epigenetic state at precise locations within the genome [45, 46] (Figure 3).

#### 3.1.4 CRISPR‐Mediated off‐Targets

CRISPR‐Cas systems can bind to unintended genomic sites and cleave DNA, leading to off‐target effects [47, 48]. These off‐target events can cause large deletions, genomic rearrangements, and lethal mutations that result in loss of gene function and may promote malignant transformation [49, 50]. Efforts to mitigate off‐target effects include enhancing on‐target efficiency and employing in silico tools for off‐target detection.

Off‐target effects occur when CRISPR mediates the excision of DNA sequences that are similar, but not identical, to the intended target, often due to sequence mismatches. It is crucial to recognise these potential risks and the ongoing strategies to minimise them [51]. Several advancements, including truncated guide RNAs (gRNAs), dual sgRNA strategies (using the nickase platform), high‐fidelity Cas9 variants, and CRISPR base editors (BE), have been developed to enhance specificity and reduce off‐target cleavage [52]. Various techniques are available for detecting off‐target effects, such as BLISS (in situ detection), LAM‐PCR HTGTS (translocation sequencing), DISCOVER‐Seq (ChIP‐based detection), GUIDE‐seq (anchored primer enrichment), CIRCLE‐seq (in vitro selection libraries), and Digenome‐seq and SITE‐Seq (in vitro genomic DNA digestion) [53, 54]. Unbiased and biased methods, such as Elevation and other detection techniques, are commonly used to identify off‐target mutations. Moreover, newly engineered Cas variants with enhanced precision have demonstrated significant reductions in off‐target activity [52].

### 3.2 Applications of CRISPR: Delivery Methods

**FIGURE 4:**
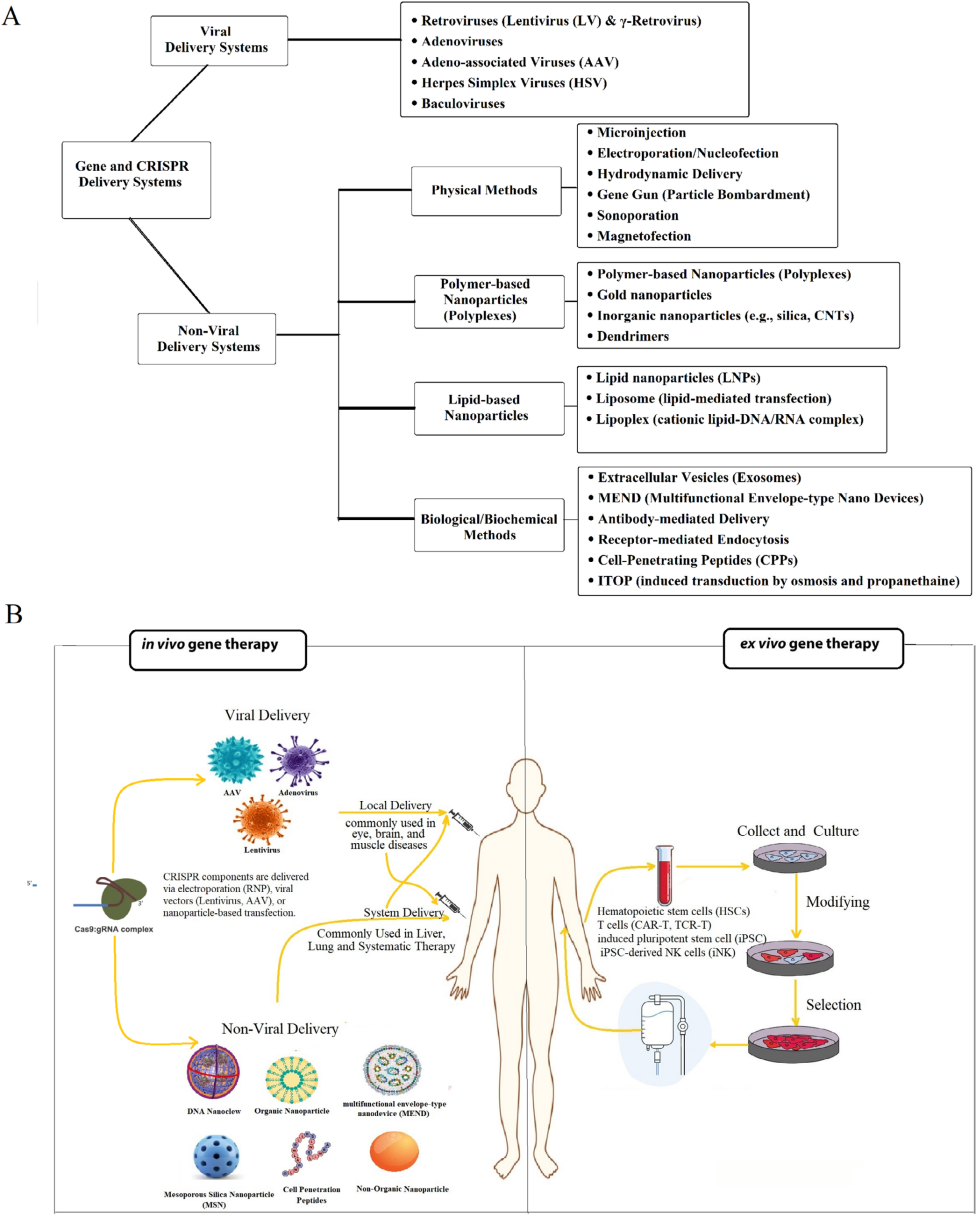
Clinical strategies and delivery platforms for CRISPR‐Cas‐based gene therapy. (A) This figure summarises key clinical applications and delivery methods for CRISPR‐Cas systems in therapeutic development. Delivery approaches are categorised into viral and non‐viral systems. Viral vectors—including lentivirus, adenovirus, adeno‐associated virus (AAV), herpes simplex virus (HSV), and baculovirus—are commonly used for their high transduction efficiency and long‐term gene expression capabilities, though each carries unique limitations in immunogenicity, cargo capacity, and integration risk. Non‐viral delivery systems are subdivided into physical (e.g., microinjection, electroporation, nucleofection, gene gun, sonoporation, hydrodynamic delivery), chemical (e.g., lipid nanoparticles, lipoplexes, gold nanoparticles, dendrimers, DNA nanoclews, inorganic nanoparticles), and biological/biochemical approaches (e.g., cell‐penetrating peptides, iTOP, receptor‐mediated endocytosis, exosomes, antibody‐based targeting). These strategies enable the delivery of CRISPR cargo in the form of plasmid DNA, mRNA, or ribonucleoprotein (RNP) complexes. The figure highlights how optimising delivery platforms is essential to overcome barriers in targeting efficiency, immune response, and in vivo applicability for clinical translation of CRISPR‐based therapies. (B) In Vivo Gene Therapy: CRISPR‐Cas systems are delivered directly into the patient using viral vectors (e.g., AAV, Lentivirus, Retrovirus) for localised (e.g., muscle) or systemic (e.g., liver, lung) gene editing. Non‐viral methods employing nanoparticles (DNA, organic, inorganic, MSN, MEND) and cell‐penetrating peptides also facilitate direct in‐body delivery. Ex Vivo Gene Therapy: Patient cells (e.g., HSCs, T‐cells, iPSC‐derived NK cells) are collected, genetically modified with CRISPR‐Cas systems in vitro, selected for desired modifications, and then re‐infused back into the patient for therapeutic effect.

Along with genome editing tools, delivery methods have been developed recently. CRISPR‐Cas9 delivery methods involve two main categories: viral and non‐viral [55]. The delivery vehicle will determine what types of DNA, mRNA, or protein can be delivered and what delivery methods achieve a safe and efficient in vivo/ex vivo delivery system for CRISPR‐Cas9 [56]. Efficient delivery of CRISPR components is critical for the success of gene editing therapies. Viral vectors such as adeno‐associated virus (AAV) and lentivirus have been widely used because of their high transduction efficiencies; however, concerns regarding immunogenicity, limited packaging capacity, and potential insertional mutagenesis remain significant challenges [55]. As depicted in Figure 4A, non‐viral delivery systems are broadly classified into physical methods (e.g., microinjection, electroporation, hydrodynamic delivery, gene gun, sonoporation, magnetofection), polymer‐based nanoparticles (e.g., polyplexes, gold and inorganic nanoparticles, dendrimers), lipid‐based nanoparticles (e.g., LNPs, liposomes, lipoplexes), and biological‐biochemical methods (e.g., exosomes, MENDs, antibody‐ and receptor‐mediated delivery, CPPs, ITOP). These platforms offer safer and more flexible alternatives to viral vectors, with reduced immunogenicity; however, their efficiency and target specificity remain ongoing challenges. Figure 4A and Table S1 [56, 57].

### 3.3 CRISPR Therapeutics: Bridging Ex Vivo Engineering and in Vivo Precision

The therapeutic strategy of CRISPR‐Cas systems could be categorised into ex vivo and in vivo. Recent advancements in engineered nanoparticles and targeted delivery strategies have improved the in vivo performance of non‐viral systems, thereby enhancing the therapeutic potential of CRISPR‐based interventions. This in‐depth understanding and optimisation of delivery methods are essential for translating CRISPR technology into clinical practice [58]. The ex vivo strategy consists of extracting and isolating targeted cells from a patient, editing them, and, as autologous transplantation, delivering the cells back to the patient [59, 60]. The summarised schematic of viral and non‐viral delivery methods is depicted in Ex vivo strategies deliver important advantages from controlled cell manipulation as well as superior quality control capabilities and the ability to increase modified cell numbers before re‐administration while presenting difficulties in achieving effective cell engraftment as well as logistical complexities during transplantation processes. The clinical advantage of direct patient delivery for CRISPR components through in vivo approaches includes quicker therapeutic action, although challenges exist regarding delivery efficiency along with immune responses and off‐target effects control. The analysis of these methods reveals their distinct advantages and disadvantages for researchers to plan upcoming discussions about particular in vivo uses [61]. In vivo, strategy is conducted by direct delivery of disease‐specific designed CRISPR‐Cas vectors to target cells or organs of the body as in situ gene therapy [57] (Figure 4B).

CRISPR‐Cas9 is being used ex vivo for cancer immunotherapy, specifically in constructing CAR‐T and TCR‐T cells [62, 63] and treating hereditary diseases such as sickle cell anaemia, β‐thalassaemia, etc. [64, 65]. Also, viral infection elimination, which is extensively and effectively used against human immunodeficiency virus (HIV), hepatitis B virus [66], and human papillomavirus (HPV) [67, 68] are engineered T cells designed to target cells for improved treatment outcomes. The main clinical application based on an in vivo strategy is monogenic disorders arising from a single‐gene defect inherited according to traditional Mendelian patterns [69]. Emerging new era treatment approaches using CRISPR to edit patient‐derived iPSCs (CRISPR iPS cells) enable scientists to correct disease‐causing mutations and a broad range of challenging diseases like cellular immunotherapies for cancer treatment and replace them by re‐introducing back into patients [70, 71]. Although the generation and initial gene editing of iPSCs are performed in vitro, the overall process is considered an ex vivo approach because the modified cells are subsequently expanded, quality‐controlled, and re‐transplanted into the patient [70] (Figure 4B).

iPSC therapy‐based treatment is classified into three main strategies: the first one is gene knock‐out, which is mainly applied to eliminate viral infections or any approach that mediates the elimination of the disease‐causing gene [72]; the second is gene knock‐in, which is an exogenous nucleotide sequence introduced to CRISPR iPS to correct a mutated gene or replace the mutated gene with the correct version of a gene/specific sequence [73]. Transcription regulation is regarded as a third strategy, which mediates transcriptional activator or suppressor of a candidate endogenous genes as a therapeutic strategy when increased or decreased expression of endogenous genes causes disease [74] (Figure 4B).

### 3.4 Miniaturised CRISPR Tools for Increased Clinical Applicability

Recent advancements in CRISPR technology have prioritised the miniaturisation of CRISPR systems to improve clinical translation by enhancing in vivo delivery and reducing immunogenicity. Compact nucleases such as Cas12f (CasMINI), CasX (also known as Cas12e), and Casθ have been engineered for their smaller size and efficient activity, making them more suitable for packaging into delivery vectors like AAVs. Researchers have improved editing performance by optimising guide RNAs—such as introducing circular guide RNAs (cgRNAs) for stability—and fusing effector domains to enable potent transcriptional regulation. CasX and Casθ, derived from non‐pathogenic bacteria, also show promising specificity and low off‐target activity, further enhancing their therapeutic potential. These developments aim to overcome the limitations of traditional CRISPR‐Cas9 systems and support broader applications in treating genetic disorders [75, 76, 77, 78].

### 3.5 Application of CRISPR in Clinic

CRISPR technology has implications for several challenging diseases, such as cancer, genetic disorders, neurologic disorders, and infectious diseases, regarding diagnosis and treatment. CRISPR‐Cas9 genome editing has been extensively investigated in preclinical and clinical settings [79].

#### 3.5.1 CRISPR Applications in Cancer: Gene Correction, Onco‐Immunotherapy, and Beyond

Cancer is a multidimensional situation comprised of environmental and intrinsic factors. Genomic mutations as a main clue in cancer pathobiology may occur as a new gene mutation, an inherited allele, or a mutation that the repair system fails to correct [80]. In any case, it causes the activation of oncogenes and/or inactivation of tumour suppressors, leading to disturbance in gene expression, the epigenome, metabolism, and eventually disruption of cell proliferation, structure, polarity, and motility [81]. In addition, such a complex situation is made by evading and circumventing the host's defence systems [82]. Gaining insights into the intricate coordination of molecular and cellular events and the consequent modifications in the tumour microenvironment is paramount in comprehending the onset, advancement, and response to therapies in diverse cancer types [83]. Such understanding serves as a cornerstone in developing innovative and more efficacious treatment approaches, with the ultimate goal of enhancing outcomes for the large number of individuals diagnosed with cancer each year [84]. It seems necessary to explore and develop a strategy to target and correct mutated genes by directly eliminating, restoring, correcting, or repairing mutated sequences/genes (Table 2) [105]. Also, an effective treatment strategy should be considered according to gene mutations and their subsequent effect on the epigenetic imprint, gene expression (based on functional mechanisms of mutated or epigenetics), corresponding protein, and molecular treatment efficacy and application [106].

**TABLE 2:** A comprehensive overview of the various approaches of treatment utilising CRISPR in cancer therapy.

Author date	Approaches	Cancer type	Main findings	Ref
Rosenblum, Gutkin et al. 2020	CRISPR Cas9 genome editing using targeted lipid nanoparticles	Glioblastoma and ovarian tumours	In vivo delivery of Cas9 mRNA and sgRNAs using CRISPR‐LNPs allows for efficient gene editing, achieving up to ~70%–80% editing. Intracerebral injection of CRISPR‐LNPs targeting PLK1 (sgPLK1‐cLNPs) in aggressive orthotopic glioblastoma resulted in tumour cell apoptosis, inhibited tumour growth by 50%, and improved survival by 30%. • Selective uptake of epidermal growth factor receptor (EGFR)‐targeted sgPLK1‐cLNPs into disseminated ovarian tumours led to inhibition of tumour growth and increased survival by 80%.	[85]
Chen, Liu et al. 2017	The CRISPR‐Cas9 system, which uses clustered regularly interspaced short palindromic repeats	Glioma	The optimised core‐shell nanostructure, liposome‐templated hydrogel nanoparticles (LHNPs), are designed to efficiently co‐deliver Cas9 protein and nucleic acids in a novel approach. LHNPs outperform Lipofectamine 2000, a commercial agent, in terms of delivery efficiency in cell culture, and can be engineered for targeted inhibition of tumour genes. LHNPs demonstrate effective inhibition of tumour growth and improved survival in tumour‐bearing mice when used for CRISPR‐Cas9 therapeutic gene targeting, such as polo‐like kinase 1 (PLK1).	[86]
Zhang, Zhang et al. 2017	Delivering a CRISPR‐Cas9 system usinPLNP‐basedPLNP based approach	Malignant melanoma, prostate cancer, and breast cancer	Over 56 agents were screened to develop a delivery system for CRISPR‐Cas9 using polyethylene glycol phospholipid‐modified cationic lipid nanoparticles (PLNP)‐a. In vitro experiments showed successful Cas9/sgPLK‐1 plasmids transfection in A375 cells using PLNP/DNA, with up to 47.4% transfection efficiency. Significant downregulation of Polo‐like kinase 1 (PLK‐1) protein and inhibition of tumour development by more than 67% were seen in in vivo investigations involving intratumor injection of Cas9/sgPLK‐1 plasmids in melanoma tumour‐bearing mice.	[87]
Behan, Iorio et al. 2018	Exploiting the stress phenotype caused by genetic changes such tumour or suppressor gene loss by the use of medications that target the oncogene or the activated downstream signalling pathway	NA	Genome‐scale CRISPR‐Cas9 screens were conducted in 204 human cancer cell lines representing 12 cancer types. A data‐driven framework was established to systematically prioritise new oncology targets based on tissue type and genotype. Werner syndrome RecQ helicase was identified as a candidate target for tumours with microsatellite instability, and its potential as a therapeutic target was verified.	[88]
Meca‐Cortés, Guerra‐Rebollo et al. 2017	Therapeutic cells and GCV	Glioblastoma	CRISPR‐Cas9‐mediated knock‐in can be a non‐viral method to introduce and stably express therapeutic genes in human amniotic mesenchymal stem cells (hAMSCs). The therapeutic potential of CRISPR‐Cas9‐engineered hAMSCs is comparable to that of therapeutic hAMSCs generated through lentiviral vector transduction with the same therapeutic gene. This approach broadly applies to other therapeutic cell modification applications, offering a versatile and efficient genetic modification strategy.	[89]
Liang, Li et al. 2017	CRISPR‐Cas9 genome editing technology, VEGFA targeting	Osteosarcoma	LC09‐functionalized PPC lipopolymer encapsulating CRISPR‐Cas9 plasmids specifically targeted VEGFA in osteosarcoma (OS) cells, decreasing VEGFA expression and secretion. This delivery system effectively inhibited the malignancy of orthotopic OS tumours, and reduced lung metastasis, angiogenesis, and bone lesions, without detectable toxicity. The delivery system simultaneously suppressed autocrine and paracrine VEGFA signalling in tumour cells, suggesting its potential for clinical translation of CRISPR‐Cas9 for cancer treatment.	[90]
Wang, Chow et al. 2019	CRISPRa mediated MAEGI	Pancreatic cancer	CRISPRa can activate the expression of multiple endogenous genes in tumours, resulting in enhanced antitumor immune responses. Precision targeting of mutated gene sets using CRISPRa can effectively eradicate a significant portion of established tumours, both locally and at distant sites. Increased T cell infiltration and immunological markers associated with anticancer activity are two outcomes of this therapy strategy that alter the tumour microenvironment.	[91]
Tang and Shrager, 2016	Surgery, radiation therapy, and/or targeted/chemotherapy	Lung cancer	CRISPR‐Cas9‐mediated genome editing is a cutting‐edge technique that enables precise modifications to cells' genomes. This technology can potentially correct or eliminate mutated EGFR genes in individual patients' cancer cells based on biopsy samples. The combination of CRISPR‐Cas9‐mediated genome editing with traditional treatment modalities such as surgery, radiation therapy, or chemo/targeted therapy could offer promising synergistic effects for cancer treatment.	[92]
Zhang, Wu et al. 2020	Gene therapy and chemotherapy	Hepatoma carcinoma (HCC)	A novel nano complex based on chitosan, functionalized with lactobionic acid and responsive to stimuli, was developed for co‐delivery of sgVEGFR2/Cas9 plasmid and paclitaxel for hepatoma carcinoma therapy. In vitro and in vivo studies showed that the nano complex achieved significant genome editing efficiency (up to 38.6% in vitro and 33.4% in vivo) of sgVEGFR2/Cas9, leading to suppression of VEGFR2 protein expression in HepG2 cells. The nano complex demonstrated potent anti‐tumour activity, inhibiting hepatoma carcinoma (HCC) tumour growth by 70% in mice. It also stimulated the anti‐tumorigenic pathway of HCC by suppressing pro‐inflammatory cytokines (IL‐6/IL‐8) and tumour angiogenesis‐related protein (NF‐κB p65) expression, indicating its potential as a therapeutic option for HCC treatment.	[93]
Zhang, Li et al. 2018	Inhibitors of B Raf proto‐oncogene, serine/threonine kinase (BRAF), inhibitors of EGFR	Melanoma, non‐small cell lung cancer	To speed up the development of heritable drug resistance models in cancer cell lines, the MED12 gene was precisely knocked out using the CRISPR‐Cas9 technology. Inhibitors of BRAF, a proto‐oncogene and serine/threonine kinase, failed to effectively treat the resulting MED12KO A375 (melanoma) cell line, whereas inhibitors of EGFR failed to effectively treat the resulting MED12KO PC9 (non‐small cell lung cancer) cell line. Combinations of BRAF inhibitors and transforming growth factor beta receptor (TGFR) inhibitors were found to be active in suppressing the growth of MED12KO A375 cells, and combinations of EGFR inhibitors and TGFR inhibitors were active in suppressing the growth of MED12KO PC9 cells; these results suggest potential therapeutic strategies for overcoming drug resistance in these cancer cell lines.	[94]
Rayner, During et al. 2019	RNP and plasmid‐based methods	Colorectal cancer (CRC)	While CRISPR‐Cas9 is a powerful tool for genome editing, there is a need for established and rigorous controls to minimise unwanted off‐target effects. False positives may occur from the inability of current methods for detecting appropriately altered clones to detect large‐scale rearrangements. To evaluate genome‐edited cells more thoroughly and reduce the possibility of off‐target effects, simple cytogenetic approaches can be used to identify clones with chromosomal abnormalities and significant mutations at the target loci.	[95]
KouranovaEvguenia, ForbesKevin et al. 2016	Column chromatography, precipitation with ethanol, and purification with 5 M ammonium sulfate all use the same volume of starting material	Glioma	CRISPR components, including Cas9 protein and guide RNA (gRNA), can be delivered into cells and single‐cell embryos using different formats, such as DNA expression plasmids, in vitro transcribed RNA, or recombinant proteins bound to the RNA portion in a ribonucleoprotein particle (RNP). RNPs, composed of Cas9 protein complexed with gRNA, are considered one of the most convenient and efficient methods for delivering CRISPR components into cells and embryos, as they can result in high editing efficiency with reduced off‐target effects. Limited off‐target effects have been detected in cells and embryos when using RNPs as a delivery method for CRISPR components, further highlighting their potential as a precise and reliable tool for genome editing applications.	[96]
Deng, Tan et al. 2019	In vivo delivery of the CRISPR Cas9 genome editing method to suppress tumour cell PD‐L1 production by knocking out the Cyclin‐dependent kinase 5 (Cdk5) gene	Murine melanoma and triple‐negative breast cancer	The cationic copolymer aPBAE was utilised to deliver CRISPR‐Cas9 genome editing technology, which was then used to knock off the Cdk5 gene in vivo. Tumour cell PD‐L1 expression was reduced after the Cdk5 gene was knocked out, indicating a possible immunomodulatory function. In murine melanoma and triple‐negative breast cancer models, Cdk5 gene knock‐out led to effective tumour growth inhibition and lung metastasis suppression, indicating the therapeutic potential of targeting Cdk5 in cancer treatment. Strong T cell‐mediated immune responses were also elicited by deleting the Cdk5 gene in the tumour microenvironment, with increased CD8+ T cells and decreased regulatory T cells (Tregs), which may account for some of the anticancer effects seen.	[97]
Szlachta, Kuscu et al. 2018	Chemotherapy and pharmacologic inhibition of MEK signalling inhibitors	Pancreatic ductal adenocarcinoma (PDAC)	Genes that show synergistic effects with MEK signalling inhibitors when deleted or inhibited were identified using CRISPR‐Cas9 genome editing technology. A novel method called Drug Response Evaluation by in vivo CRISPR Screening (DREBIC) was developed to evaluate drug response in cancer cells, and its efficacy was validated using large‐scale experimental data from independent experiments. DREBIC demonstrated high accuracy in predicting drug response in cancer cells from diverse tissues, and it identified therapeutic vulnerabilities of cancer‐causing mutations to MEK inhibitors in various cancer types. The findings suggest that targeting specific genes in combination with MEK signalling inhibitors could potentially enhance the cytotoxicity of these inhibitors and provide new therapeutic strategies for cancer treatment.	[98]
Lin, Larrue et al. 2020	NA	Acute myeloid leukaemia (AML)	Orthotopic xenograft animals were used to construct a CRISPR screening strategy for validating and prioritising AML‐enriched dependencies in vivo. Several targets with translational value were identified, including SLC5A3 as a metabolic vulnerability and MARCH5 as a critical guardian to prevent apoptosis in AML. It was demonstrated that repression of MARCH5 enhanced the efficacy of BCL2 inhibitors, such as venetoclax, suggesting the clinical potential of targeting MARCH5 in AML. • These findings provide insights into potential therapeutic targets for AML treatment and highlight the importance of in vivo CRISPR screening approaches for identifying relevant dependencies in cancer models.	[99]
Liu, Zhao et al. 2018	Gene delivery of CRISPR‐Cas9 system with nanocarriers	Chronic myeloid leukaemia (CML)	The CRISPR‐Cas9 system, with its gRNA targeting the BCR‐ABL gene, holds the potential for developing novel therapeutics for CML. Polymeric nanoparticles (CLANs) loaded with the CRISPR‐Cas9 plasmid (pCas9/gBCR‐ABL) can specifically disrupt the CML‐related BCR‐ABL gene while sparing the normal BCR and ABL genes in healthy cells. Intravenous injection of CLANpCas9/gBCR‐ABL in a CML mouse model improved survival, suggesting that combining the CRISPR‐Cas9 system with nanocarriers is a promising strategy for targeted treatment of CML. This approach has the potential to provide a more precise and effective therapeutic option for CML patients, minimising off‐target effects and reducing harm to normal cells. Further research and development in this area could lead to the development of novel therapies for CML and other cancers.	[100]
Cheung, Chow et al. 2018	Antibody‐based targeted therapy and synthetic lethality	Lung cancer	The L858R point mutation in EGFR was targeted with the CRISPR‐Cas9 system by utilising an oligonucleotide guide that detects the mutation as the proper PAM sequence for DNA cleavage. This strategy demonstrated high specificity, resulting in selective genome cleavage, specifically in cells harbouring the L858R mutation. Treated cells with the L858R mutation exhibited reduced EGFR expression and decreased cell proliferation, resulting in a smaller tumour load in vivo. This study highlights the potential of CRISPR‐Cas9‐mediated genome editing as a precise approach for targeting specific mutations, such as the L858R mutation in EGFR, and modulating gene expression and cellular function. Further research and development in this area could lead to developing novel therapeutic strategies for cancers and other genetic diseases associated with specific mutations, providing new treatment options for patients.	[101]
Wang and Sun 2017	CRISPR‐Cas9‐mediated targeting of the oncogene HER2	Breast cancer	Inhibition of cell proliferation in breast cancer cell lines was achieved by using the CRISPR‐Cas9 technology to directly target the oncogene HER2. By inhibiting poly ADP‐ribose polymerase, the inhibitory effect of CRISPR‐mediated HER2 targeting was amplified. Interestingly, the CRISPR‐induced mutations did not significantly affect the level of HER2 protein expression but instead exerted its effect through a dominant negative mutation, likely disrupting HER2 signalling or function. These findings highlight the potential of CRISPR‐Cas9‐mediated genome editing as a promising approach for targeting oncogenes, such as HER2, in cancer cells.	[102]
Ibrahim, Özsöz et al. 2019	Editing, modifying, deleting, adding, and replacing DNA sequences	NA	CRISPR‐Cas9 and CRISPRi are genetic editing tools used for modifying DNA sequences. CRISPR‐Cas9 induces a double‐strand break and uses HDR to introduce changes in DNA. CRISPRi uses a modified Cas9 protein to block gene expression by preventing transcription. CRISPRi is reversible and tunable, allowing for control of gene expression without permanent changes to DNA. Both CRISPR‐Cas9 and CRISPRi have diverse applications in research, drug discovery, and gene therapy, but ethical considerations are important for their responsible use.	[103]
Cook and Ventura 2019	Immuno oncology therapeutic approaches involving engineered cell‐based therapy using CRISPR‐based approaches	Non‐small cell lung cancer	CRISPR‐based cancer therapeutics and diagnostics are advancing rapidly and expected to significantly impact clinical practice in the near future. CRISPR‐engineered chimeric antigen receptor‐T (CAR‐T) cells are being developed to create safer and more effective therapies for immunologically ‘cold’ tumours. Innovative modifications of CRISPR enzymes enable rapid, simple, and sensitive detection of specific nucleotide sequences for diagnostics and molecular diagnostics applications.	[104]

*Note*: The table covers the cancer type that each approach is targeting, as well as the main findings of each study. Cancer therapy has been an area of intense research, and CRISPR technology has emerged as a promising tool for precision cancer treatment.

**FIGURE 5:**
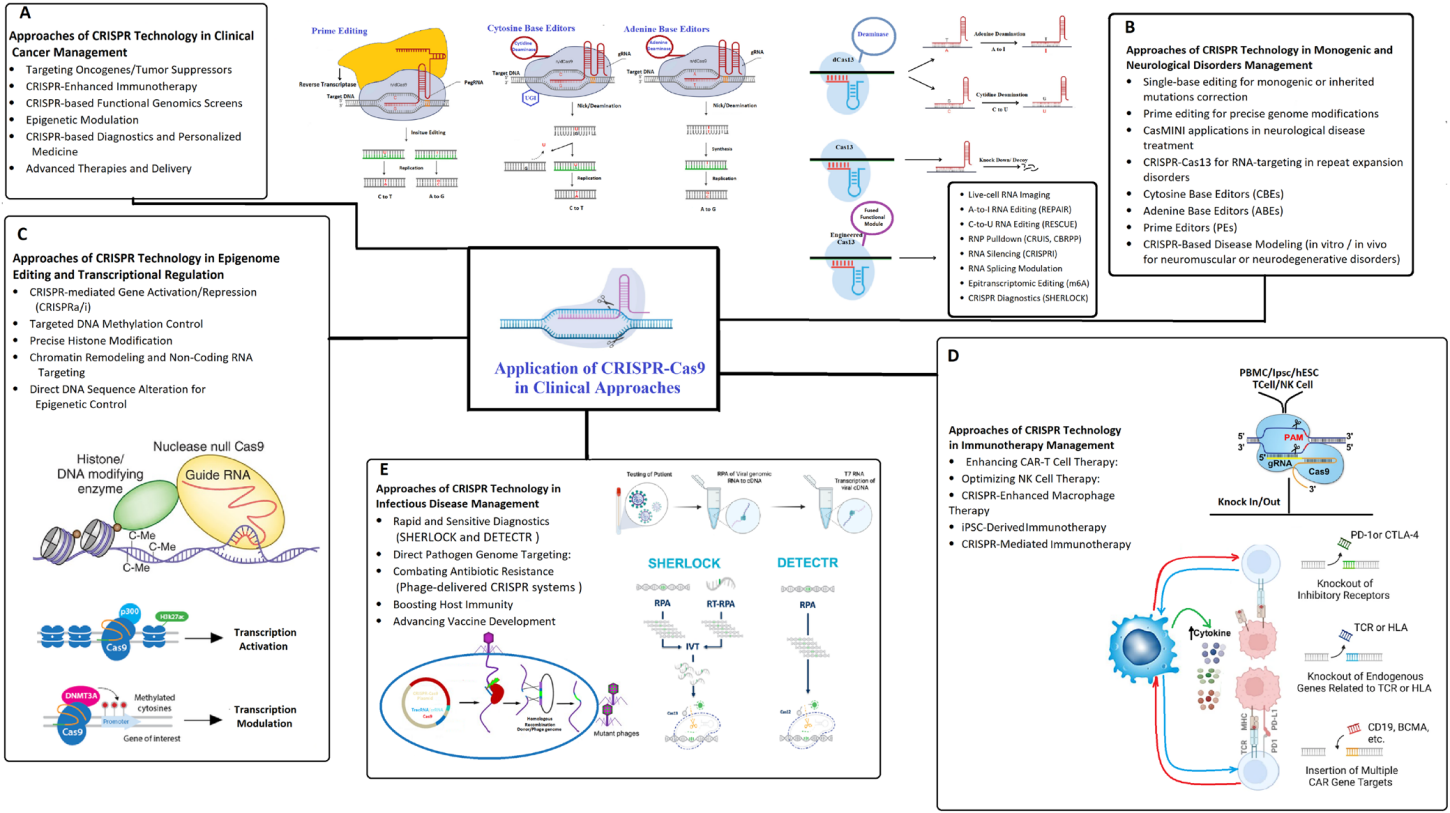
CRISPR‐Cas applications in clinical disease management and epigenetic regulation. This figure highlights the diverse and expanding clinical applications of CRISPR‐Cas technologies across five major therapeutic areas. In the cancer section, CRISPR is used to disrupt oncogenes, enhance immunotherapy (e.g., editing PD‐1 or TCRs), perform functional genomic screens to identify vulnerabilities, and support precision medicine strategies. In immunotherapy, CRISPR enables engineered T cells, CAR‐T cell modification, and checkpoint inhibition to improve immune targeting of tumours. The epigenome editing section illustrates CRISPR‐based gene regulation techniques such as CRISPR interference (CRISPRi) and activation (CRISPRa), targeted methylation via DNMT3A, and histone modifications through fusion with epigenetic enzymes. Precise transcriptional modulation is achieved without altering the DNA sequence. The histone methylation/demethylation area includes Cas‐fusion systems that specifically target histone markers, enabling dynamic chromatin remodelling. In infectious disease applications, CRISPR systems are employed for pathogen genome editing, antiviral strategies, and diagnostics using SHERLOCK/DETECTR platforms. Additionally, the lower portion of the figure illustrates emerging genome editing strategies such as prime editing and base editing, showing how cytosine and adenine base editors, along with reverse transcriptase‐guided pegRNAs, enable single‐nucleotide modifications without DSBs. This multi‐tiered representation underscores CRISPR's therapeutic versatility from genomic modification to epigenetic tuning and pathogen detection.

The concern of gene mutation behind cancer pathobiology caused the emergence of CRISPR‐Cas9 as a powerful method for making desired changes and corrections [107, 108]. CRISPR‐Cas9‐mediated established cells and animal models have resulted in clinical trials, which potentially treat tumours and offer great promise for inhibiting migration, invasion, and even treatment of tumours [107, 109, 110]. Some of the most clinically advanced strategies built from CRISPR systems present themselves in the form of immunotherapies which utilise CAR‐T and CAT‐T‐cell therapies. This method utilises CRISPR technology to improve cancer‐targeting functions in immune cells while providing additional therapeutic possibilities to direct gene editing [63]. CRISPR‐based CAR‐T therapies involve engineering T cells to express synthetic receptors targeting tumour antigens, often incorporating gene edits like PD‐1 or TCR knockouts to enhance efficacy and reduce exhaustion. In contrast, CRISPR‐altered T‐cell (CAT‐T) therapies focus on modifying endogenous T cells for CAT‐T therapies typically involves using gene editing tools like CRISPR‐Cas9 to alter their natural T‐cell receptors (TCRs). This process aims to redirect the T cells' inherent ability to recognise specific cancer antigens, often those presented by human leukocyte antigen (HLA) molecules, including patient‐specific neoantigens. The modification can involve inserting new TCR alpha and beta chains that confer desired specificity, which then complex with the existing CD3 components to form a functional, tumour‐targeting TCR [111, 112]. While CAT‐T approaches may offer improved safety profiles, their reliance on native TCR specificity can limit efficacy, particularly in tumours with low immunogenicity. Both strategies leverage CRISPR's precision to optimise therapeutic outcomes, with CAR‐T therapies demonstrating significant success in hematologic malignancies, and CAT‐T approaches being explored for solid tumours and other applications. Ongoing research continues to refine these modalities, aiming to balance efficacy, safety, and accessibility in cancer immunotherapy [107, 113]. The main approaches to treatment using CRISPR in cancer therapy studies are represented in Table 2 and schematically in Figure 5A.

#### 3.5.2 CRISPR‐Enhanced Immunotherapy: Engineering T Cells and NK Cells for Precise Medicine

Chimeric Antigen Receptor (CAR) T‐cell therapy, a ‘living drug’ using genetically engineered patient T cells to target and eliminate cancer, has revolutionised blood cancer treatment [114]. CRISPR‐Cas9 significantly enhances CAR‐T outcomes by enabling precise CAR gene integration, mitigating T‐cell exhaustion through knockout of inhibitory genes (e.g., PD‐1, CTLA‐4, and LAG‐3), and improving CAR‐T survival and anti‐cancer effects by interfering with programmed cell death [115, 116, 117, 118]. While T‐cell exhaustion remains a challenge, immune checkpoint inhibitors do not perpetually resolve CAR‐T drawbacks. CRISPR also facilitates “off‐the‐shelf” allogeneic CAR‐T development by removing endogenous TCRs to prevent GVHD and deleting MHC molecules to avoid host rejection [115, 116, 117, 119].

Besides disrupting inhibitory receptor removal, CRISPR‐Cas9 can interfere with the interaction between Fas and Fas ligands responsible for programmed cell death, increasing CAR‐T cells' anti‐cancer effect. Importantly, CRISPR‐Cas9 has a signature in removing the CAR‐T cell resistance [118]. The successful development of iPSC‐derived CAR‐engineered T (iCART) cells capable of eliminating cancer cells represents a pivotal advancement in clinical translation and therapeutic application (Figure 5D) [120].

CRISPR‐Cas9 has played a pivotal role in increasing the efficacy of NK (natural killer) cells in immunotherapy against AML [120]. NK cells, which are significant lymphocytes in the innate immune system, can be engineered using CRISPR‐Cas9 to knock out immune checkpoints and inhibitory signals, integrate tissue homing receptors to improve tumour infiltration, provide extra activating signalling to enhance anti‐tumour activity, and arm with chimeric antigen receptors (CARs) to enhance tumour specificity. CRISPR‐Cas9 can also be used to transcriptionally activate genes, such as the NKG2D ligand MICA, to improve the activation of NK cells [121]. In addition, CRISPR‐Cas9‐mediated disruption of PD‐1 and targeting of other immunosuppressive pathways, such as the adenosine 2A receptor, has been shown to enhance the efficacy of CAR‐T (chimeric antigen receptor‐T cell) therapy against cancer [122]. Furthermore, CRISPR‐Cas9‐mediated targeting of the Cish gene has been shown to enhance the cytotoxicity of primary human NK cells towards B lymphoma cells, demonstrating the potential of CRISPR in improving NK cell‐based immunotherapies [123, 124].

Modified NK cells generated using CRISPR‐Cas9 can express various receptors, such as high‐affinity DC16 Fc receptor or CAR receptors, which can improve their potency against solid tumours when combined with other therapies [125, 126]. Studies have shown the feasibility and efficacy of using induced pluripotent stem cells (iPSCs) as a platform to generate NK cells carrying cancer‐homing CAR receptors (CARiPSC‐NK cells), which demonstrated impressive anti‐tumour activity in ovarian cancer xenograft models with improved cell survival and expansion in vivo and less toxicity [127]. The FDA recently approved using off‐the‐shelf NK cells produced from clonal master iPSCs, named FT500, which synergized with T cells to treat solid tumour malignancies more effectively [128, 129] (Figure 5D).

For autoimmune conditions like systemic lupus erythematosus, cellular immunotherapies such as CD19‐targeted Chimeric Antigen Receptor (CAR) T cells are being explored to redirect immune cells and correct underlying autoimmunity by selectively eliminating pathogenic B cells, aiming for potential single‐administration cures [130, 131]. Building on these advancements, precision engineering of T cells, notably through CRISPR‐based chimeric autoantigen‐T cell receptors (CATCR), offers an even more refined approach. CATCR‐T cells are designed to reprogram a patient's T cells to specifically seek out and bind to receptors on autoimmune disease‐causing B cells, leading to their selective elimination while safeguarding normal immune function [132]. This innovative strategy provides tailored treatments that can control autoimmunity without the broad infection risks associated with conventional immunosuppressive therapies, marking a significant step towards bespoke solutions for devastating autoimmune conditions [133].

To ensure reproducibility and translational potential, studies utilising human stem cell‐derived immunotherapies, such as iPSC‐derived NK or CAR‐T cells, should adhere to the International Society for Stem Cell Research (ISSCR) guidelines. These standards, endorsed by journals such as *Cell Proliferation*, provide comprehensive recommendations for the characterisation, quality control, and ethical use of human pluripotent and tissue stem cells in research and clinical applications [134, 135].

#### 3.5.3 Targeting Monogenic Diseases With CRISPR: From Bench to FDA‐Approved Therapies

The CRISPR‐based gene treatments for monogenic disorders have shown major clinical advancements that demonstrate their power for transformation. The breakthrough achievement in gene therapy emerged when the Food and Drug Administration (FDA) approved CRISPR‐based treatment CTX001 to treat sickle cell disease in the United States. The CRISPR‐based therapy CTX001 changes patient‐specific haematopoietic stem cells in a laboratory for fetal haemoglobin reactivation to solve disease symptoms while making clinical problems less severe [136]. Clinicians are currently studying CRISPR‐based therapeutic methods for genetic (inherited) diseases beyond sickle cell and beta‐thalassaemia as specific cases to prove the far‐reaching medical possibilities of this technology for treating genetic‐based disorders (Table 3).

**TABLE 3:** An overview of the various approaches of treatment utilising CRISPR in Common Genetic Disorders.

Disease	Monogenic or polygenic/target gene	Stem cell	Cell line	In vivo model	Transfer model	Ref
SCA	Monogenic/β‐globin	hiPSCs	HEK293T, bc1, TNC1	Human	Electroporation, transfection	[137]
PV	Monogenic/JAK2	hiPSCs	HEK293T	Human	Transfection	[138]
β‐ Thalassaemia	Monogenic/HBB gene	hiPSCs		Human	Transfection	[139]
Cataracts	Monogenic/Crygc	SSCs		Mouse	Microinjection, transfection, electroporation	[140]
A1AT deficiency	Monogenic/SERPINA1	hiPSCs	HEK293T	Human	Transfection	[138]
FA	Monogenic/FANCC		Patient fibroblasts	Human	Transfection	[141]
PKU	Monogenic/PAH		c.1222C>T COS‐7	Human	Transfection	[142]
CF	Monogenic/CFTR	Intestine Stem cell	HEK293T	Human	Transfection	[143]
UCD	Monogenic/OTC		MC57G	Mouse	Transfection	[144]
AHC and HH	Monogenic/DAX1			Monkey	Microinjection	[145]
HT‐I	Monogenic/Fah	3 T3 cells	Mouse		Hydrodynamic injection	[146]
Osteosarcoma	Monogenic/CDK11		KHOS and U‐20S	Human	Transfection, electroporation	[147]
CRC	Polygenic/APC, SMAD4, TP53, KRAS, PIK3CA		Human intestinal epithelial organoids	Human	Transfection	[148]
Bronchial alveolar adenoma	Polygenic/Kras, p53, Lkb1	mESCs	Neuro‐2a	Mouse	Transfection	[149]
CVD	Monogenic/Pcsk9		3 T3‐L1	Mouse	Transfection	[150]
Barth syndrome	Monogenic/TAZ	hiPSCs	PGP1 cell line	Human	Transfection	[151]
Intestinal hyperplasia	Monogenic/Apc	mESCs		Mouse	Blastocycst injection	[152]
Cardiomyopathy	Monogenic/Myh6		10 T1/2 cells	Mouse	Intraperitoneal injection	[153]
DS	Monogenic/GATA 1		K562	Human	Transfection	[154]
RP	Monogenic/RGPR	iPSCs		Human	Transfection	[155]
Contextual fear memory	Monogenic/Mecp2		HEK293FT	Mouse	Transfection Stereotactical injection	[156]
DMD	Exon 45 of dystrophin gene, Exon 23 of dystrophin gene (Monogenic)	iPSC	Myoblasts, Muscle stem cells, Immortalised patient myoblasts, HEK293T, C2C12 and mdx	Mice, Human	Microinjection, nanoparticle, transfection, electroporation, lipofection	[157, 158, 159, 160, 161, 162]
ALS and/or FTD	Polygenic/SOD, FUS, CHMP2B, Ku80, C9ORF72	iPSC	LCL, FUS/TLS cell line	Human, G93A‐SOD1 transgenic mice	Electroporation, nucleofection, Adeno‐associated virus, transfection	[163, 164, 165, 166, 167, 168]
Dravet Sundrome	Monogenic/Scnla	Mouse embryonic hippocampal neurons	P‐19	Mice	Adeno‐associated virus	[169]
SCID X‐1	Monogenic/IL2Rg	hESCs	K‐562, T hCD4+cells	Human	Transfection	[170]

Abbreviations: AD, Alzheimer disease; AHC, Adrenal hypoplasia congenital; CF, cystic fibrosis; CRC, colorectal cancer; CVD, cardiovascular disease; DMD, Duchenne muscular dystrophy; DS, Down syndrome; DrS, Dravet syndrome; FA, Fanconi anaemia; HD, Parkinson disease; HH, hypogonadotropic hypogonadism; HT1, hereditary tyrosinemia type I; PD, Parkinson Disease; PKU; PhenylKetonUria; PV, Polycythemia Vera; RP, retinitis pigmentosa; SCA, sickle cell anaemia; SCID, severe combined immune deficiency; UCD, urea cycle disorder.

Clinical genetic disorder management is divided into two main approaches: pre‐ and post‐genomic. In pre‐genomic procedures, single‐base gene editing mediated by CRISPR is a hallmark, while when an original gene is damaged and inactivated, the inactivation of a single deleterious gene cannot address the complex situation of multifactorial disease [125]. Therefore, newly developed single‐base substitution tools, such as cytosine BE, ABE, and prime editors (PEs), could partially compensate for the limitations. In the era of post‐genomics, CRISPR‐Cas9 technology has been employed to develop new versions of chimer Cas protein to explore intended genes in genome splicing [171], transcription [172], modification [173], and epigenetic regulation [174] (Figure 5B).

#### 3.5.4 CRISPR Against Infectious Diseases: Diagnostic and Therapeutic Frontiers

HIV, Hepatitis B, human papillomavirus, and recently, COVID‐19 are among the more challenging viruses, increasing healthcare concerns and socioeconomic burdens. Due to immune evasion, mutation, latency, and chronic infection leading to organ damage, fighting against some types of viruses is more challenging for new medical strategies. In this regard, CRISPR technology, as a unique gene‐editing tool, offers a novel medical approach to diagnosing and predicting threats of infectious diseases. The two analogous diagnostic systems, DETECTR [175, 176] and SHERLOCK [177, 178], have been designed to detect RNA and DNA sequences, respectively. Following the outbreak of COVID‐19 infection, besides real‐time polymerase chain reaction (RT‐PCR) and the Loop‐mediated isothermal amplification (LAMP) method, which are the most applied diagnostic methods for detecting the virus RNA [179]. Also, CRISPR‐based detection methods rapidly emerged as highly sensitive alternatives to RT‐PCR and LAMP, enabling cost‐effective and rapid diagnoses of SARS‐CoV‐2 [180, 181]. Additionally, CRISPR‐based therapeutic approaches have been developed to selectively target and eradicate viral and bacterial genomes [182] (Figure 5E).

CRISPR‐Cas9 is employed to target essential viral genes such as the long terminal repeats, CCR5, and CXCR4 of the HIV virus, utilising single or dual‐guide RNAs to enhance editing efficiency [183]. Targeting viral replication factors like BST‐2/tetherin and promoter regions has further improved viral suppression [184, 185, 186, 187, 188, 189, 190]. Similarly, CRISPR‐Cas9 has demonstrated efficacy in disrupting the oncogenes E6 and E7 of high‐risk HPV types (HPV‐16 and HPV‐18) [191] leading to cell growth inhibition and apoptosis induction [192] with ongoing research extending to low‐risk HPV types (e.g., HPV‐6/11) and vaccine development [193]. Moreover, CRISPR‐Cas13, targeting RNA, has been investigated for its antiviral activity against SARS‐CoV‐2, effectively inhibiting viral replication in human respiratory cells [194]. Several studies were conducted to explore the anti‐COVID‐19 effect of CRISPR. It is documented that the CRISPR‐ased antiviral modality potently inhibits viral replication and activity in human respiratory epithelial cells and cleaves 100% of positive RNA viruses [195] (Table 4).

**TABLE 4:** An overview of the various approaches of treatment utilising CRISPR in common viral disease.

Disease	Target gene	Stem cell	Cell line	In vivo model	Transfer model	References
HIV‐1 resistance	CCR5	hiPSCs	HEK293T	Human	Transfection	[196]
HIV‐1 infection and immunisation	LTR loci of integrated viral genome, T10	J‐lat	CHME5	Human	Transfection	[197]
EBV	Multiple –gene EBV nuclear antigen 1 (EBNA‐1)		Ranji and Burkitt's lymphoma cell lines	Human	Transfection	[198]
HPV and cervical cancer	HPV16, E7 oncogenes		SiHa and Caski	Human	Transfection	[199]
HBV	p53 and Pten gene		Huh7, HepG2	Mouse	Transfection, electroporation hydrodynamic Injection	[200, 201]
Cryptosporidiosis	*C. parvum*		HTC8	Mouse	Transfection	[202]
Herpes Simplex 1 (HSV‐1)	Multiple genes		Vero cells human oligodendroglioma cells	Human	Transfection	[203]
Human herpesvirus 6 (HHV‐6)	Eradication the viral genome trough targeting conserved region of the DRs		293 T cell line harbouring the integrated virus genome (293 T‐6A) & iciHHV‐6A patient cells	Human	Transfection	[204]
Human cytomegalovirus (CMV)		Immediate‐early (IE) genes	HFF primary fibroblasts THP‐1 monocytic cell line	Human	Transfection	[205]
JC polyomavirus		JCV T‐antigen	HJC‐2 cells	Human	Transfection	[206]
SARS‐CoV‐2		RdRP / N gene regions	Cas13d A549 cells huMAN cells (PAC‐MAN)	Human	Transfection	[195]

Abbreviations: DrS, Dravet syndrome; EBV, Epstein–Barr virus; HBV, hepatitis B virus; HIV, human immunodeficiency virus; HPV, human papillomavirus.

Besides HIV and HPV infection, CRISPR was recruited to eliminate other well‐known DNA‐based viruses, including *Epstein–Barr virus* (EBV), *cytomegalovirus*, *Sars‐cov‐2*, JC virus, and *Zika* virus [207, 208] (Table 4). Although CRISPR technology is derived from bacteria, it has been used against bacterial infections such as *Acinetobacter baumannii*, *Escherichia coli* , and *Staphylococcus aureus* ‐induced osteomyelitis by eliminating infectious agents and resolving drug resistance [209, 210, 211].

#### 3.5.5 Application of CRISPR in Neurological Disorders

Genetic mutations are responsible for several neurological diseases, and gene editing approaches are currently used to diminish the burden of neurological disease [212]. Although disease‐modifying therapies were approved for some neurological disorders, there is a lack of disease‐modifying therapies for some other conditions, and clinicians have tried symptom therapy [213]. Although CRISPR‐Cas9 was applied to set up neurological disease animal models, CRISPR targeting neurological disorder strategies is currently used to treat neurological diseases in the clinical setting (Figure 5B) [214] (Table 5). The initial CRISPR‐Cas9 application in neurological disease was presented in the monogenic condition, where CRISPR was used in Duchenne muscular dystrophy (DMD) [227]. Subsequent studies focused on other monogenic disorders of the nervous system, such as spinal muscular atrophy (SMA), Parkinson's disease (PD), amyotrophic lateral sclerosis (ALS), Huntington's disease (HD), and Alzheimer's disease (AD) in both in vitro and in vivo settings [228]. Moreover, CRISPR can also be applied to hereditary neurological disorders like Down syndrome, fragile X syndrome, Tay‐Sachs, Sandhoff, and Niemann‐Pick, well‐known as incurable diseases (Table 5).

**TABLE 5:** A comprehensive overview of the various approaches of treatment utilising CRISPR in neurological disorders.

Disease (abbreviations)	Target gene	Delivery method & model	Stem cell	Cell line	Animal model	References
AD	APP, PSEN2, Gmf, Bacel	Transfection, Adeno‐associated virus, nucleofection, nanocomplexes	Fibroblast, iPSCs	Primary cortical neurons and hippocampus of Tg2576 mice, BV2 cells, CA3 hippocampus	AD patients, mice	[215, 216, 217]
ALS and/or FTD	SOD, FUS, CHMP2B, Ku80, C9ORF72	Electroporation, nucleofection, Adeno‐associated virus, transfection	iPSC	G93A‐SOD1, C9ORF72‐ALS/FTD	Human, mice	[163, 164, 165, 166, 167, 168]
Ds	Scnla	Adeno‐associated virus			DS mice	[169]
Epilepsy	Kcnal (Monogenic)	Adeno‐associated virus			Camk2a‐Cre mice and epileptic mice	[218]
HD	HTT gene (Monogenic)	Stereotactic injection, lentivirus, Adeno‐associated virus	BM‐MSCs	HEK293T	Mouse	[219, 220, 221, 222] , [157]
PD	SNCA, hM4Di, hM3Dq, mcu, sRAGE, DJ1, parkin, PINK1, LRRK2 (Polygenic)	Lentivirus, Adeno‐associated virus, microinjection	iPSCS, UCB‐MSCs, MD NPCs, zebrafish, Procrine fetal fibroblasts		Human, zebrafish, pig	[223, 224, 225]
Dyslexia	DIP2A (Monogenic)	PGK‐Puro‐P2A‐mCherry	homozygous knockout 46C ESC cell line		Murine ES cell	[226]

#### 3.5.6 Prospects of CRISPR‐Mediated Epigenetic Therapeutic Interferences

Epigenetic modifications of genomic DNA and histone proteins, such as methylation or acetylation, at specific genomic loci or histone residues, have been shown to play critical roles in biological fates.

The emergence of the CRISPR‐Cas9 system led to access to DNA and histones for epigenetic editing. Utilising HNH/UvC Dead Cas9 as a DNA‐binding domain, fused to enzymes such as DNA methylases, histone acetyltransferases/deacetylases, and histone methyltransferases/demethylases can be targeted to alter the epigenetic state at precise locations (Figure 5C) [174].

Recruitment of epigenome editing effector domains utilising CRISPR‐Cas systems allows for the stimulation and repression of endogenous gene expression and graded control of gene regulation [229]. Fusing chromatin‐modifying domains enables targeted epigenome editing of nuclease‐deactivated Cas9 (dCas9) in cultured cells and animal models. On the other hand, delivering large dCas9 fusion proteins to desired tissues and target cells is an obstacle for in vivo studies.

For example, a nuclease‐dead Cas9 fused to the p300 histone acetyltransferase domain (dCas9–p300) has been shown to target H3K27 acetylation at specific loci, driving robust transcriptional activation; in transgenic mouse models, gRNA‐targeted dCas9–p300 altered epigenetic states and downstream gene expression in the brain and liver in vivo [230]. Notably, burn injury activates a histone H3.1 Ser10 phosphorylation signal in spinal dynorphinergic (Pdyn) neurons, and CRISPR‐Cas9‐mediated S10 → A mutagenesis of H3.1 in these neurons (via AAV delivery) blocked this modification and significantly elevated thermal nociceptive thresholds in mice [231]. Complementary CRISPR modalities also enable targeted genetic modulation in tissue trauma: base editors allow precise single‐base corrections without double‐strand breaks, and dCas9–KRAB or Cas9 nuclease strategies can respectively repress or knock out genes, facilitating gene silencing or knock‐in approaches for pain and tissue‐damage pathways (Figure 5C) [232].

#### 3.5.7 Current Landscape of CRISPR‐Based Therapeutics in Clinical Applications: Recent Approvals and Ongoing Trials

CRISPR gene editing has reached significant clinical milestones as of 2025, and several promising treatments have emerged from trials. The first CRISPR–Cas9–based cell treatment for sickle cell disease (SCD) was exagamglogene autotemcel (Casgevy), which was licenced in late 2023 [233]. In early 2024, it was approved for transfusion‐dependent β thalassaemia [234]. This ex vivo therapy edits a patient's haematopoietic stem cells to induce fetal haemoglobin, and 94% of treated SCD patients achieved freedom from vaso‐occlusive crises for at least 1 year [233]. Beyond haemoglobin disorders, in vivo CRISPR therapies are advancing rapidly. Transthyretin amyloid cardiomyopathy (ATTR‐CM) can be treated with NTLA‐2001, a one‐time intravenous CRISPR treatment that has advanced to a pivotal Phase 3 trial after showing > 90% reduction in pathogenic TTR protein levels in patients [235] and clinical metrics indicating disease stabilisation. Similar to this, NTLA‐2002, an in vivo CRISPR therapy for hereditary angioedema, reduced attack rates by 91%–97% with a single dosage [236] enabling patients to remain attack‐free, with no side effects reported, paving the way for a Phase 3 study.

CRISPR‐engineered cell therapies are also showing promise in oncology. In its Phase 1 trial, a CRISPR‐edited allogeneic CAR T‐cell treatment (CB‐010) for recurrent B‐cell lymphoma produced a 94% overall response rate, including 69% total complete remissions [237]. The first‐ever durable full remission (lasting more than 3 years) in solid tumours was achieved with an off‐the‐shelf CAR‐T method using CRISPR‐edited T cell therapy (CTX130) that targets CD70 in advanced renal cell carcinoma [238]. This result highlights the potential for gene‐edited immune cells to tackle solid cancers, and next‐generation versions (e.g., CTX131) are already under development. Beyond traditional CRISPR nucleases, base‐editing approaches have entered the clinic: a base editor therapy (VERVE‐101) for heterozygous familial hypercholesterolemia demonstrated substantial LDL cholesterol reductions (50% lower sustained at 6 months) after a one‐time treatment [239], marking the first proof‐of‐concept for base editing in humans. Even infectious diseases are being targeted—a CRISPR‐based therapy aimed at curing HIV (EBT‐101) has shown that the approach is feasible and safe in early trials, although viral rebound occurred after stopping antiretroviral therapy (8), underscoring the challenges ahead.

This finding underscores how gene‐edited immune cells can take on solid cancers, and next‐generation versions (e.g., CTX131) are in the pipeline. In addition to conventional CRISPR nucleases, base‐editing strategies have now been brought into the clinic: a base editing therapy (VERVE‐101) for heterozygous familial hypercholesterolemia showed robust reductions in LDL cholesterol (50% decrease at 6 months after a single treatment) [239], representing the first proof‐of‐principle for base editing in humans. Even infectious diseases are being targeted—a CRISPR‐based therapy for the cure of HIV (EBT‐101) has been shown to be both feasible and safe in early trials, but has resulted in viral rebound following discontinuation of ART [240] Several clinical trials have explored the impact of CRISPR in patients with cancer. This article mentions the recent (from 2018) clinical trials documented at ClinicalTrials.gov in Table S2.

#### 3.5.8 Diagnosis

Beyond its therapeutic potential, CRISPR‐Cas9 holds significant promise in diagnostics, offering rapid, sensitive, specific, accurate, and cost‐effective detection for pathogens, early cancer diagnosis, single nucleotide polymorphisms, and genetic diseases. Notable platforms include SHERLOCK (Specific High‐sensitivity Enzymatic Reporter unlocking), utilising Cas13 for highly sensitive and specific RNA detection in viral infections (e.g., Zika, dengue) and cancer‐related mutations [241, 242, 243]. DETECTR (DNA Endonuclease‐Targeted CRISPR Trans Reporter) is another CRISPR‐based diagnostic technique that uses the Cas12 enzyme to detect specific DNA sequences. DETECTR is a particular and sensitive tool for detecting mutations associated with genetic diseases, such as sickle cell anaemia, and identifying specific strains of bacteria [244]. These CRISPR‐based diagnostics offer a powerful alternative to traditional cancer screening methods, which often lack the necessary sensitivity and speed for early detection, potentially improving patient prognoses [245]. In colorectal cancer (CRC), where early detection significantly improves outcomes, CRISPR‐Cas9 shows promise in identifying key gene mutations (e.g., PIC3C3A, KRAS, TP53, SMAD4, APC) in CRC organoids and detecting de novo driver genes. Furthermore, CRISPR‐Cas13 technology offers a superior alternative to qRT‐PCR for detecting tumour‐derived microRNAs (e.g., miR‐23a, miR‐1290, miR‐126, and miR‐940), overcoming the limitations of traditional methods for early CRC diagnosis [246].

Besides its use in early diagnosis of CRC, CRISPR‐Cas9 can be applied as a screening modality to determine de novo diagnostic biomarkers like proteins, genes, and receptors involved in the cell signalling of pancreatic cancer cells, including drug sensitivity, apoptosis, proliferation, and spheroid formation [247]. For example, the overexpression of the HDAC1 gene induces epithelial‐to‐mesenchymal transition (EMT), a well‐established mechanism contributing to chemoresistance [248]. Similarly, the PRMT5 gene has been implicated in the development of chemoresistance; conversely, its inhibition renders pancreatic cancer cells more susceptible to gemcitabine, a commonly used first‐ or second‐line chemotherapeutic agent [249]. Approximately 130 genes are responsible for platinum resistance in pancreatic cancer cells [250]. The CRISPR‐Cas system represents a transformative approach for both curing and diagnosing pancreatic cancer, particularly in overcoming platinum resistance [251, 252]. Previous research reveals that CRISPR/Cas9 screening effectively identifies key genes, such as BRIP1, ERCC4, and FANCD2, alongside pathways enriched in DNA binding and NADH dehydrogenase activity, which modulate sensitivity to platinum‐based chemotherapies [251, 253]. By precisely editing these resistance‐conferring genes, CRISPR can synergistically enhance the efficacy of chemotherapy, for example, by disrupting oncogenic KRAS mutations or restoring tumour suppressor functions like TP53 [254]. In diagnostics, CRISPR‐based platforms like SPEAR offer highly sensitive and rapid detection of critical cancer‐related mutations, such as KRAS, from liquid biopsies, enabling earlier diagnosis and disease monitoring [255]. Despite its immense promise, challenges remain, notably the delivery of CRISPR components through the dense desmoplastic stroma of pancreatic tumours, although strategies like iRGD‐guided tumour‐penetrating nanocomplexes and stroma‐modulating agents are being explored to overcome this barrier [256, 257]. This integration of CRISPR‐mediated gene modification with personalised screening using patient‐derived organoids is paving the way for tailored, more effective treatment strategies against this aggressive cancer.

CRISPR can be utilised as a powerful detector of specific bacterial pathogens since CRISPR is one of the bacterial defence systems [258, 259]. Still, the diagnostic properties are not limited to only diagnosing pathogenetic bacteria. CRISPR can also be utilised to diagnose viral pathogens. The gold standard for diagnosing SARS‐CoV‐2 during the recent pandemic is the reverse transcription‐quantitative polymerase chain reaction (RT‐qPCR). However, this method has significant drawbacks, including its confinement to specialised laboratories, the requirement for skilled workers, and the possibility of erroneous or misleading data [260]. CRISPR‐based next‐generation diagnostic technologies offer a solution to these issues; they are affordable, sensitive, specific, and do not necessitate complex equipment so that they may be employed in settings outside of the clinical field [261].

#### 3.5.9 Challenges and Limitations CRISPR Bench to Bedside

Technology like the CRISPR‐Cas9 platform allows reverse mutagenesis, which could be used to treat disease [262]. To effectively manipulate the genome to remove and replace (Knock‐in) causative mutations or generate host alterations via insertion or deletion as protective functions is what is meant by the term ‘targeted gene therapy’ [58]. First, HDR to NHEJ must be overcome to replace a gene sequence with a provided fragment encoding the necessary repair to generate protein variants with healthy phenotypes. In the latter case, NHEJ is a crucial part of the HIV ongoing evaluation because it effectively generates a nonfunctional allele of the gene [55, 262]. Despite the treatment strategy, the complexity of the gene therapy candidate disease is another challenge in finding effective treatments [263]. In contrast to monogenic disorders, which can be easily treated by correcting a faulty copy of the culprit gene, polygenic diseases, and cancers require numerous simultaneous modifications to the genome to accomplish a successful treatment [69]. Another important challenge in gene therapy is that efficient and effective correction occurs at all cell cycle stages [264]. The efficacy of gene therapy depends on several factors, including the disease's genetic background and topologies of the target and pattern sequences, the type of therapeutic alteration, the repair mechanism intended to be recruited, the effectiveness of delivery techniques, and the cell state [265]. Therefore, before moving on to human trials, all therapeutic ideas must be proved in model organisms to address these problems and propose solutions [266]. All these challenges can be different from one person to another, which is the main description of personalised medicine. Even though the preceding explanation suggests that CRISPR‐Cas9 is a practical method, this gene editing system has various limitations that make its application in clinical trials problematic due to its recent identification and use in patients. A few approaches have been explored to address the CRISPR‐Cas9 system's drawbacks, including immunogenicity, off‐targeting, polymorphism, delivery technique, and ethics [17].

Some challenges prevent the widespread utilisation of CRISPR‐Cas9 in cancer research and therapies, the most significant of which is the technique's limited editing efficacy in tumours and the potential toxicity of existing delivery vehicles. To successfully implement genome editing systems in the intended species or cells, CRISPR‐Cas9 delivery relies on the availability of adequate and effective alternatives of delivery mechanisms. Unfortunately, getting the Cas9 system into living organisms has been difficult until now. The Cas9‐based gene editing platform has been delivered by integrating physical approaches with viral vectors. In vitro, the physical methods are more practical than the viral vector‐based methods, which often have low packing capacities and a high risk of side effects. Non‐viral drug delivery technologies, including polymeric and lipid nanocarriers, have shown promising delivery performance and targeting efficacy in recent preclinical and animal investigations to deliver CRISPR‐Cas9 systems. These adeno‐associated viruses are regarded as possible gene editing system transporters. The efforts to modify charged nanocarriers by altering their structures are described, and prospective non‐viral vectors undergoing clinical studies are highlighted. Delivering CRISPR‐Cas molecules into target cells is essential for therapeutic purposes, which have been facing difficulties since transfer vesicles can be affected by immune reactions, toxicity, or rapid degradation. Extracellular vesicles (EVs), a wide array of membrane vesicles, have been proposed as a hypothetical replacement. Many cells can generate EVs; exosomes are the smallest, with only 40–15 nm diameters, and are released by endocytes [267, 268]. Exosomes are carriers of molecules such as messenger RNA (mRNA) and miRNA, which can be absorbed by target cells through various forms of endocytosis or fusion with the plasma membrane [269]. This natural affinity of exosomes provides a significant opportunity to act as carriers of intercellular nucleic acids and therapeutic molecules, thus bypassing the aforementioned difficulties in transferring CRISPR‐Cas molecules into targets through biological membranes [270]. The CRISPR‐Cas9 technology, which can be directly applied to embryos, requires less time to implement compared to gene‐targeting technologies that use embryonic stem cells (ES cells). Very robust processes that guarantee the introduction of the intended mutation are now possible, thanks to advances in bioinformatics tools (to find the most acceptable sequences to build guide RNAs) and in optimising experimental settings.

DNA fragments (such as cDNAs) can be inserted into a genome using Cas9 to generate a DSB and activate the DNA repair machinery. Targeted alleles often have additional alterations, such as deletions, partial or multiple incorporations of the targeted vectors, or even duplications, because the DNA repair system's objective is not to insert DNA pieces into the genome. Even while secondary unwanted mutational events at the target locus plague conventional ES cell‐based experiments, researchers have found a means to avoid creating mice with passenger mutations. Most research facilities employ positive and negative selection procedures, as well as validation methods that seek out further mutations in the target location, to isolate ES cells that have undergone the desired recombination events. However, the CRISPR‐Cas9 strategy cannot select the intended goal when applied to embryos, greatly reducing the likelihood of identifying the target allele. Because of the observed mosaicism in CRISPR‐Cas9‐created founder mice, it is especially challenging to discover unwanted genomic modifications around the targeted region [271].

## 4 Closures and Conclusion

CRISPR‐Cas9 genome editing technology created a fundamental transformation in medical science that enables DNA modification through site‐specific programs with extraordinary precision and minimal difficulty. The clinical and research applications of CRISPR systems expand quickly but multiple essential obstacles need resolution to achieve their therapeutic maximum. The priority now is to boost CRISPR platform accuracy since minimal off‐target events can generate major implications during medical applications. Different upcoming genome editing tools including Cas12a, Cas13, base editors and prime editors demonstrate potential to enhance both specificity and genome editing capability. Predictive algorithms together with machine learning‐guided gRNA design techniques provide essential tools to decrease off‐target activities. The deployment of genome editing remains the main bottleneck that stands in the way of its use for translation. The refinement of both viral vectors as well as non‐viral delivery vehicles (lipid nanoparticles and polymeric systems) continues but tissue‐specific scalable delivery systems functioning in a transient manner represent the upcoming priority. The combination of nanotechnology and synthetic biology has demonstrated potential to create specialised solutions for dealing with these problems. Research demonstrates that CRISPR shows rising applicability in treating complex diseases like malignancies in addition to neurodegenerative conditions and autoimmune disorders and monogenic disorders. New therapeutic opportunities emerge for polygenic diseases by implementing combination gene editing approaches along with epigenetic therapy methods such as dCas9‐p300 and dCas9‐KRAB and CRISPR‐based on/off control tools. Social and ethical along with regulatory issues possess similar importance to the field. The development and implementation of socially responsible policies require interdisciplinary teams to address problems related to germline editing and CRISPR‐based therapy availability as well as long‐term medical safety. Visual engagement between stakeholders becomes essential to guarantee proper clinical implementation with ethical standards. Future progress in CRISPR depend on combining it with AI‐powered target identification systems which pair with high‐throughput single‐cell screening methods and intelligent delivery platforms to become a fundamental precision medicine platform. The next 10 years will determine whether CRISPR advances from groundbreaking technology into the everyday medical practice for curing difficult‐to‐treat diseases.

## Author Contributions

Bahareh Farasati Far and Sherko Nasseri contributed to the conception and design of the study. Material preparation and data collection were conducted by Sherko Nasseri, Mohammad Amin Habibi, and Bahareh Farasati Far. All authors participated in drafting the initial version of the manuscript. Sherko Nasseri redesigned and rewrote the manuscript, including the design of five figures in alignment with the revised content. During the revision process, Bahareh Farasati Far, Marziyeh Akbari, Morteza Katavand, and Sherko Nasseri contributed to all manuscript modifications. All authors reviewed and approved the final version of the manuscript. Sherko Nasseri supervised all steps of manuscript preparation, revision, and submission.

## Ethics Statement

The authors have nothing to report.

## Consent

The authors have nothing to report.

## Conflicts of Interest

The authors declare no conflicts of interest.

## Supporting information


**Table S1.** Overview of viral and non‐viral CRISPR delivery methods and their characteristics. This table summarises a broad range of non‐viral delivery systems developed for transporting CRISPR components—including DNA, RNA, and ribonucleoprotein (RNP) complexes—into target cells. The methods are categorised by their underlying mechanisms (physical or chemical), and each entry details the delivery approach, cargo type, major advantages, and associated drawbacks. Technologies include well‐established platforms like electroporation and lipofection, as well as emerging nanotechnologies such as gold nanoparticles, DNA nanoclews, and multifunctional envelope‐type nano devices (MENDs). The table emphasises each method’s utility for in vitro or in vivo applications, editing efficiency, cytotoxicity, delivery specificity, and translational feasibility. This comparative overview supports the selection of optimal non‐viral delivery strategies in CRISPR‐based research and therapeutic development.

**Table S2.** Ongoing clinical trials and applications of CRISPR. Summary of significant clinical developments and recent studies using CRISPR‐based therapies. As of early 2025, this table lists notable instances of CRISPR technology applications through clinical trials. In a variety of disease areas, such as genetic disorders, oncology, cardiovascular disease, and infectious disease, it highlights a range of therapeutic approaches, such as ex vivo cell therapies, in vivo gene editing via nucleases base editors, and engineered cell therapies. Key reported discoveries or outcomes, the target indication, the current clinical trial phase, the particular therapy or application, and pertinent citations are all included in the details.

## Data Availability

The data that support the findings of this study are available from the corresponding author upon reasonable request.
