# Supplementary material for: CRISPR Technology in Disease Management: An Updated Review of Clinical Translation and Therapeutic Potential
Source: Cell Prolif. 2025 Jul 20;58(11):e70099. doi: 10.1111/cpr.70099 (PMC12584872; doi:10.1111/cpr.70099)
Supplement: Supplementary file 1 — Table S1. Overview of viral and non‐viral CRISPR delivery methods and their characteristics. This table summarises a broad range of non‐viral delivery systems developed for transporting CRISPR components—including DNA, RNA, and ribonucleoprotein (RNP) complexes—into target cells. The methods are categorised by their underlying mechanisms (physical or chemical), and each entry details the delivery approach, cargo type, major advantages, and associated drawbacks. Technologies include well‐established platforms like electroporation and lipofection, as well as emerging nanotechnologies such as gold nanoparticles, DNA nanoclews, and multifunctional envelope‐type nano devices (MENDs). The table emphasises each method’s utility for in vitro or in vivo applications, editing efficiency, cytotoxicity, delivery specificity, and translational feasibility. This comparative overview supports the selection of optimal non‐viral delivery strategies in CRISPR‐based research and therapeutic development. [file CPR-58-e70099-s001.docx]

**Table Supplementary 1. Overview of Viral and Non-Viral CRISPR Delivery Methods and Their Characteristics.**

This table summarizes a broad range of non-viral delivery systems developed for transporting CRISPR components—including DNA, RNA, and ribonucleoprotein (RNP) complexes—into target cells. The methods are categorized by their underlying mechanisms (physical or chemical), and each entry details the delivery approach, cargo type, major advantages, and associated drawbacks. Technologies include well-established platforms like electroporation and lipofection, as well as emerging nanotechnologies such as gold nanoparticles, DNA nanoclews, and multifunctional envelope-type nano devices (MENDs). The table emphasizes each method's utility for in vitro or in vivo applications, editing efficiency, cytotoxicity, delivery specificity, and translational feasibility. This comparative overview supports the selection of optimal non-viral delivery strategies in CRISPR-based research and therapeutic development.

| Method | Category | Approach | CRISPR Cargo | Advantages | Disadvantages |
| --- | --- | --- | --- | --- | --- |
| Electroporation | Non-viral​ | Physical | DNA, RNA, or RNP​ | High transfection efficiency; broad cell-type applicability (in vitro & in vivo); supports plasmid, mRNA, or RNP delivery​ | Induces significant cell toxicity (high cell death); non-specific (affects most cells); very low stable integration of delivered DNA (~0.01%)​ |
| Microinjection | Non-viral​ | Physical | DNA, RNA, or RNP​ | Precise single-cell delivery with high specificity; applicable to plasmid DNA, mRNA, or pre-formed Cas9 RNP complexes​ | Labor-intensive and low-throughput (one cell at a time); can damage cells (requires skilled operation)​ |
| Nucleofection | Non-viral | Physical | DNA or RNP​ | \|  \| \| --- \|   Direct nuclear delivery of cargo  (via specialized electrical pulse),  which is especially useful for  hard-to-transfect primary cells ;  high editing efficiency (similar to  Electroporation). | \|  \| \| --- \|   Similar drawbacks to standard electroporation (cell stress and death)​; requires specialized electroporation equipment and optimization for each cell type. |
| Hydrodynamic delivery | Non-viral​ | Physical | DNA (plasmid)​ | \| Simple, rapid injection method for in vivo gene transfer; **highly efficient for liver** gene delivery in small animals​; effective for  plasmid-based CRISPR systems  in mice. \| \| --- \| | \| can cause severe physiological  stress (cardiac overload, liver  damage)​; not suitable for large  animals or clinical use​; mainly  effective for liver (less so for  other organs)​ \| \| --- \| |
| CPPs (Cell-penetrating peptides) | Non-viral | Chemical | Nucleoprotein (Cas9 RNP)​ | Small peptide carriers that penetrate cell membranes; generally safe and low toxicity​  ; bypass endocytosis by direct translocation. | Requires chemical conjugation of the peptide to the Cas9 protein or RNP​; delivery efficiency may be lower than other methods (requires co-delivery of sgRNA if not pre-complexed). |
| MENDs (Multifunctional Envelope-type Nano Devices) | \| Non-viral \| \| --- \| | \| Non-viral \| \| --- \| | \| Non-viral \| \| --- \| | Greatly enhances DNA delivery efficiency vs. naked DNA (lipid–polymer envelope boosts uptake by ~10–1000×)​; versatile design – lipid envelope can be modified with PEG, targeting ligands, endosomal escape peptides, etc., to improve delivery​ | Multi-component formulation (condensed DNA + polymer + lipid) is complex; so far limited to in vitro research, with no in vivo CRISPR demonstrations yet |
| DNA nanoclew | Non-viral | Chemical | Nucleoprotein (Cas9 RNP) | Programmable DNA nanosphere that binds Cas9 RNP via base-pairing to the sgRNA, enabling high genome-editing efficiency (comparable to viral methods)​  ; tunable size and structure for optimization​; entirely virus-free components. | Requires elaborate self-assembly (rolling circle amplification)​  ; DNA carrier can be unstable (susceptible to nucleases)​  and may trigger immune sensors​  ; not yet widely tested in vivo. |
| Gold nanoparticle | Non-viral | Chemical | Nucleoprotein (Cas9 RNP)​ | Efficient at delivering Cas9 RNP into cells (high knockout rates)​  ; gold core is inert and easily functionalized (can attach multiple RNPs or targeting ligands) while being minimally immunogenic​ | Potential cytotoxicity at high particle concentrations​; can induce immune responses (e.g. cytokine production) if not properly surface-coated​  ; non-degradable, so particles may accumulate in organs. |
| Inorganic nanoparticles (e.g. silica, CNTs) | Non-viral | Chemical | Inorganic nanoparticles (e.g. silica, CNTs) | Uniform, tunable nanocarriers that are easy to synthesize with controlled size and surface chemistry​; highly stable and non-biological (no viral genes); can be chemically functionalized for targeting. | Still experimental for CRISPR (few successful reports to date)​  ; non-biodegradable particles risk accumulation in vivo; typically require additional surface modifications for effective cellular uptake. |
| iTOP (induced transduction by osmocytosis and propanebetaine) | Non-viral | Physical | Nucleoprotein (Cas9 RNP)​ | Utilizes a chemical osmotic shock to induce macropinocytosis, allowing effective Cas9 RNP uptake (~70% gene KO reported in human cells)​; no virus or special carrier needed (CPP-independent method). | Limited to in vitro use; lower efficiency in certain cells (especially primary cells)​; not suitable in vivo because it requires high salt/hyperosmolar conditions​ |
| Lipid nanoparticles (LNPs) | Non-viral | Chemical | DNA, RNA, or RNP​ | Biocompatible nanocarriers that protect nucleic acids from degradation and deliver them into cells via endocytosis​; easy to produce and scalable​; amenable to in vivo use (some LNP formulations are in clinical trials for RNA drugs)​; no cargo size limitations. | Transfection efficiency typically lower than viral vectors​; may require optimized lipid formulations or chemical tweaks for large Cas9 RNP payloads​; potential immune activation or toxicity depends on lipid components. |
| Liposome (lipid-mediated transfection) | Non-viral | Chemical | DNA or RNA​ | Simple, widely-used method for gene transfection in vitro (e.g. Lipofectamine kits)​; can effectively deliver plasmids or mRNA to many cell lines; encapsulates and protects the genetic cargo; relatively low cytotoxicity in vitro. | Limited efficiency in hard-to-transfect cells or in vivo​; often trapped in endosomes without additional release strategies; cationic liposomes at high doses can be toxic and have reduced efficacy in the presence of serum. |
| Lipoplex (cationic lipid–DNA/RNA complex) | Non-viral | Chemical | DNA or RNA​ | Forms spontaneously by mixing cationic lipids with nucleic acids; shields DNA/RNA from nucleases and can promote endosomal escape; capable of delivering large plasmids (>9 kb) with appropriate lipid formulations​ | Transfection success depends on lipid composition (inefficient with suboptimal lipids)​; primarily effective in cell culture – serum in vivo can destabilize complexes; cationic lipid toxicity is a concern if dosage is high. |
| L-MSN (Lipid-coated mesoporous silica NP) | Non-viral | Chemical | DNA, RNA, or RNP | Hybrid nanoparticle with a porous silica core (high loading capacity for large DNA or protein/RNP cargos) and a lipid shell for biocompatibility​; customizable pore size and surface chemistry to suit different CRISPR components; lipid coating can be tailored for targeting and endosomal escape. | Not yet demonstrated for CRISPR delivery in practice (still a developing approach)​  ; packaging very large biomolecules (Cas9 RNP or plasmid) is challenging​  ; long-term fate of silica core in vivo is unclear (potential accumulation if not degradable). |
| Gene Gun (Particle Bombardment) | Non-viral | Physical | DNA or RNA | High penetration into tough tissues like skin or plant cells; direct physical delivery | Low targeting precision; limited use in mammalian cells; risk of tissue damage |
| Sonoporation | Non-viral | Physical | DNA, RNA, or RNP | Non-invasive; can target deep tissues using ultrasound-guided delivery | Low reproducibility; possible cellular/tissue damage |
| Magnetofection | Non-viral | Physical | DNA or RNA (attached to magnetic particles) | Magnet-guided localization; useful for targeted delivery in vitro | Requires magnetic nanoparticles; limited in vivo efficacy |
| Dendrimers | Non-viral | Chemical | DNA or RNA | High cargo loading capacity; tunable surface chemistry for targeting | Potential toxicity; still in experimental stages for CRISPR delivery |
| Extracellular Vesicles (Exosomes) | Non-viral | Biochemical | DNA, RNA, or RNP | Natural delivery vesicles; low immunogenicity; potential for tissue-specific targeting | Difficult to produce in large quantities; heterogeneity of vesicles |
| Antibody-Mediated Delivery | Non-viral | Biochemical | Typically Cas9 protein or RNPs | High specificity via antibody–antigen targeting; potential for cell-type–specific delivery | Requires conjugation methods; limited clinical validation |
| Receptor-Mediated Endocytosis | Non-viral | Biochemical | Often Cas9 RNP or nanoparticles | Cell-specific uptake; biologically relevant targeting | Efficiency depends on receptor expression; endosomal trapping may occur |
| Lentivirus | Viral | Integrating (retroviral) | DNA | High transduction efficiency; stable integration leading to long-term expression; broad tropism (infects dividing and non-dividing cells) | Potential insertional mutagenesis (oncogenesis risk); derived from HIV (safety concerns) |
| Adenovirus | Viral | Non-integrating (episomal) | DNA | High transduction efficiency; broad host range (infects dividing and non-dividing cells); large packaging capacity | Strong immunogenicity; typically only transient expression (no integration); historical toxicity concerns |
| AAV (Adeno-associated virus) | Viral | Non-integrating (episomal) | DNA | Minimal pathogenicity; mild immune response; long-term expression (especially in non-dividing cells) | Very small packaging capacity (~4.7 kb); pre-existing anti-AAV immunity; difficult large-scale production |
| HSV (Herpes Simplex Virus) | Viral | Non-integrating (episomal) | DNA | Extremely large cargo capacity; natural neurotropism; broad host tropism; lifelong episomal persistence | Cytotoxicity; typically short-term expression unless latent; strong immune responses |
| Baculovirus | Viral | Non-integrating (episomal) | DNA | Very large cargo capacity (≥38 kb); broad tropism; inherently nontoxic in humans; easy scalable production | Inactivated by serum complement; transient expression; limited in vivo persistence |
