# Supplementary material for: CRISPR Technology in Disease Management: An Updated Review of Clinical Translation and Therapeutic Potential
Source: Cell Prolif. 2025 Jul 20;58(11):e70099. doi: 10.1111/cpr.70099 (PMC12584872; doi:10.1111/cpr.70099)
Supplement: Supplementary file 2 — Table S2. Ongoing clinical trials and applications of CRISPR. Summary of significant clinical developments and recent studies using CRISPR‐based therapies. As of early 2025, this table lists notable instances of CRISPR technology applications through clinical trials. In a variety of disease areas, such as genetic disorders, oncology, cardiovascular disease, and infectious disease, it highlights a range of therapeutic approaches, such as ex vivo cell therapies, in vivo gene editing via nucleases base editors, and engineered cell therapies. Key reported discoveries or outcomes, the target indication, the current clinical trial phase, the particular therapy or application, and pertinent citations are all included in the details. [file CPR-58-e70099-s002.docx]

**Table Supplementary 2. Ongoing Clinical Trials and Applications of CRISPR**Summary of Significant Clinical Developments and Recent Studies Using CRISPR-Based Therapies. As of early 2025, this table lists notable instances of CRISPR technology applications through clinical trials. In a variety of disease areas, such as genetic disorders, oncology, cardiovascular disease, and infectious disease, it highlights a range of therapeutic approaches, such as *ex vivo* cell therapies, *in vivo* gene editing via nucleases base editors, and engineered cell therapies. Key reported discoveries or outcomes, the target indication, the current clinical trial phase, the particular therapy or application, and pertinent citations are all included in the details.

| Therapeutic Disease | Application (Therapy) | Clinical Trial Phase | Key Development (Outcomes) | References |
| --- | --- | --- | --- | --- |
| Sickle Cell Disease (SCD) | *Ex vivo* CRISPR-edited autologous HSC therapy (Casgevy) | Phase 3 (Completed; Approved) | ~94% of patients achieved durable remission (no vaso-occlusive crises for ≥12 months) ([FDA Approves First Gene Therapies to Treat Patients with Sickle Cell Disease | [1, 2] |
| Beta Thalassemia (Transfusion-Dependent) | *Ex vivo* CRISPR-edited autologous HSC therapy (Casgevy) | Phase 3 (Completed; Approved) | High rate of transfusion independence achieved in clinical trials, leading to approval in the US/EU in 2024) (first CRISPR therapy for TDT) | [1] |
| Transthyretin Amyloidosis (ATTR-CM) | NTLA-2001 *in vivo* CRISPR-Cas9 gene knockout (IV infusion) | Phase 1 (Completed; Phase 3 ongoing) | Single-dose in vivo genome editing achieved ~90% mean reduction in serum TTR protein, with clinical stabilization of cardiomyopathy; first in vivo CRISPR therapy to advance to Phase 3 | [3] |
| Hereditary Angioedema (HAE) | NTLA-2002 *in vivo* CRISPR-Cas9 gene knockout (IV infusion) | Phase 1/2 | Single dose led to 91–97% reduction in plasma kallikrein and attack frequency, with no severe adverse events; potential functional cure for HAE, pivotal trial in planning | [4] |
| Relapsed/Refractory B-cell Lymphoma | CB-010 allogeneic anti-CD19 CAR T (CRISPR-edited T cells) | Phase 1 | 94% overall response rate and 69% complete remission in Phase 1 | [5] |
| Metastatic Clear Cell Renal Carcinoma (RCC) | CTX130 allogeneic anti-CD70 CAR T (CRISPR-edited T cells) | Phase 1 | Achieved a durable complete remission ≥3 years; 81% disease control rate observed, supporting development of next-gen CTX131 | [6] |
| Leber Congenital Amaurosis type 10 (LCA10) | EDIT-101 in vivo CRISPR-Cas9 gene editing (subretinal injection) | Phase 1/2 | First in vivo CRISPR trial in humans; 3 of 14 treated patients showed meaningful vision improvement (↑ visual acuity) with no ocular serious adverse events (proof-of-concept achieved) | [7] |
| Familial Hypercholesterolemia (HeFH) | VERVE-101 *in vivo* base-editor (adenine base edit in PCSK9) | Phase 1 | First clinical base-editing therapy; single-dose treatment produced ~50–55% reduction in LDL cholesterol at 6 months post-edit, demonstrating durable gene editing in patients | [8] |
| HIV-1 Infection (chronic) | EBT-101 *in vivo* dual-gRNA CRISPR (AAV-delivered gene therapy) | Phase 1/2 | First CRISPR-based approach aiming for HIV cure; demonstrated safety and viral vector distribution in early patients, but HIV rebound occurred after stopping ART in an initial cohort | [9] |

**References:**

1. Parums, D.V., *First regulatory approvals for CRISPR-Cas9 therapeutic gene editing for sickle cell disease and transfusion-dependent β-thalassemia.* Medical science monitor: international medical journal of experimental and clinical research, 2024. **30**: p. e944204-1.

2. Food, U. and D. Administration, *FDA approves first gene therapies to treat patients with sickle cell disease.* FDA <https://www>. fda. gov/news-events/press-announcements/fda-approves-first-gene-therapies-treat-patients-sickle-cell-disease (8 December 2023), 2023.

3. Kotit, S., *Lessons from the first-in-human in vivo CRISPR/Cas9 editing of the TTR gene by NTLA-2001 trial in patients with transthyretin amyloidosis with cardiomyopathy.* Global Cardiology Science & Practice, 2023. **2023**(1): p. e202304.

4. Longhurst, H.J., et al., *CRISPR-Cas9 in vivo gene editing of KLKB1 for hereditary angioedema.* New England Journal of Medicine, 2024. **390**(5): p. 432-441.

5. O'Brien, S., et al., *A first-in-Human phase 1, multicenter, open-label study of CB-010, a next-generation CRISPR-edited allogeneic anti-CD19 CAR-T cell therapy with a PD-1 knockout, in patients with Relapsed/Refractory b cell non-Hodgkin lymphoma (ANTLER study).* Blood, 2022. **140**(Supplement 1): p. 9457-9458.

6. Pal, S.K., et al., *CD70-targeted allogeneic CAR T-cell therapy for advanced clear cell renal cell carcinoma.* Cancer discovery, 2024. **14**(7): p. 1176-1189.

7. Chiu, W., et al., *An update on gene therapy for inherited retinal dystrophy: experience in Leber congenital amaurosis clinical trials.* International journal of molecular sciences, 2021. **22**(9): p. 4534.

8. Horie, T. and K. Ono, *VERVE-101: a promising CRISPR-based gene editing therapy that reduces LDL-C and PCSK9 levels in HeFH patients*. 2024, Oxford University Press.

9. *CRISPR gene therapy EBT-101 does not prevent HIV viral rebound.* 2024 [cited 2025 Apr 16]. Available from: <https://www.aidsmap.com/news/may-2024/crispr-gene-therapy-ebt-101-does-not-prevent-hiv-viral-rebound>.
